# Comparative profiling of MAIT cells across gastrointestinal cancers identifies conserved NK receptor dysregulation and HCC-specific defects in cytotoxicity and XBP1s signaling

**DOI:** 10.3389/fimmu.2026.1924000

**Published:** 2026-10-05

**Authors:** Vanessa Proß, Annkathrin Helena Ruhm, Linda Marie Laura Thole, Laura Tóth, Gishnu Harikumar Parvathy, Pia Jäger, Christina Götz, Georg Damm, Daniel Seehofer, Christian Bayerl, Hermann Herbst, Alexander Macke, Verena Frese, Akio Sakaki, Marlene Hanisch-Hernandez, Friederike Rosseck, Sascha Treskatsch, Frank Friedersdorff, Christoph Treese, Severin Daum, Dominik Laue, Benjamin Ruf, Sören Lukassen, Katja Kotsch

**Affiliations:** 1 Department of General and Visceral Surgery, Charité - Universitätsmedizin Berlin, Campus Benjamin Franklin, Corporate Member of Freie Universität Berlin, Humboldt-Universität zu Berlin, and Berlin Institute of Health, Berlin, Germany; 2 Department of Hepatobiliary Surgery and Visceral Transplantation, Clinic for Visceral, Transplant, Thoracic and Vascular Surgery, Leipzig University Medical Center, Leipzig, Germany; 3 Department of Radiology, Charité - Universitätsmedizin Berlin, Campus Benjamin Franklin, Corporate Member of Freie Universität Berlin, Humboldt-Universität zu Berlin, and Berlin Institute of Health, Berlin, Germany; 4 Department of Pathology, Vivantes Klinikum Neukölln, Berlin, Germany; 5 Department of Visceral Surgery, Vivantes Klinikum Neukölln, Berlin, Germany; 6 Department of Pathology, Charité - Universitätsmedizin Berlin, Corporate Member of Freie Universität Berlin, Humboldt-Universität zu Berlin, and Berlin Institute of Health, Berlin, Germany; 7 Department of Anesthesiology and Intensive Care Medicine, Charité - Universitätsmedizin Berlin, Corporate Member of Freie Universität Berlin and Humboldt-Universität zu Berlin, Campus Benjamin Franklin, Berlin, Germany; 8 Department of Urology, Evangelisches Krankenhaus Königin Elisabeth Herzberge, Berlin, Germany; 9 Medical Department, Division of Gastroenterology, Infectious Diseases and Rheumatology, Charité - Universitätsmedizin Berlin, corporate member of Freie Universität Berlin, Humboldt-Universität zu Berlin, and Berlin Institute of Health, Berlin, Germany; 10 Department of Traumatology and Reconstructive Surgery, Campus Benjamin Franklin, Charité - Universitätsmedizin Berlin, Corporate Member of Freie Universität Berlin, Humboldt-Universität zu Berlin, and Berlin Institute of Health, Berlin, Germany; 11 Department of Internal Medicine I, University Hospital Tübingen, Eberhard Karls University of Tübingen, Tübingen, Germany; 12 Center of Digital Health, Berlin Institute of Health at Charité - Universitätsmedizin Berlin, Berlin, Germany

**Keywords:** cytotoxicity, HCC, mucosa-associated invariant T cells, natural killer cell receptors, NKG2D, XBP1s

## Abstract

**Background & aims:**

Mucosal-associated invariant T (MAIT) cells are innate-like T lymphocytes with cytotoxic capacity and emerging roles in tumour immunity. While MAIT cells express a diverse repertoire of natural killer (NK) receptors, the functional significance of these receptors in gastrointestinal (GI) cancers remains poorly understood.

**Methods:**

We characterized the NK receptor repertoire and effector function of MAIT cells across five human GI tumour entities: hepatocellular carcinoma (HCC; n = 30), colorectal liver metastases (CRLM; n = 15), colorectal cancer (CRC; n = 17), pancreatic ductal adenocarcinoma (PDAC; n = 17), and gastric cancer (GC; n = 18), and examined the functional phenotype and cytotoxic potential of primary HCC-derived MAIT cells.

**Results:**

MAIT cell frequencies varied across tumour entities and anatomical compartments, with significant intratumoral depletion in HCC compared with matched peritumoral tissue. Across all tumour entities, tumour-infiltrating MAIT cells consistently exhibited a pronounced downregulation of the activating NK receptor NKG2D. This reduction was also evident in peripheral blood MAIT cells but notably reversed following tumour resection and correlated with clinical outcomes. Functionally, IL-15-induced cytotoxicity was impaired in HCC-derived MAIT cells independently of receptor expression, suggesting additional mechanisms of dysfunction. Mechanistically, we report the expression of the endoplasmic reticulum stress-responsive transcription factor X-box binding protein 1 XBP1s (spliced form of XBP1) in circulating and tissue-resident MAIT cells. XBP1s expression was induced by IL-15 and ER stress but was attenuated in tumour-derived compared with peritumoral MAIT cells, indicating defective ER stress signalling.

**Conclusions:**

Together, these findings define a distinct dysfunctional state of tumour-resident MAIT cells in HCC, characterized by defective ER stress responses, and identify impaired NK receptor expression across GI cancers that may contribute to impaired antitumour immunity.

## Introduction

Gastrointestinal (GI) tumours represent a major global health burden, accounting for more than one-third of cancer-related deaths worldwide (1). Mucosal-associated invariant T (MAIT) cells are abundant in the gastrointestinal tract and have emerged as important contributors to cancer immunity (2, 3). They are characterized by expression of a semi-invariant T cell receptor (TCR) α-chain (Vα7.2) and recognize microbial-derived vitamin B metabolites presented by the highly conserved MHC class I–like molecule MR1 (4, 5). MAIT cells are terminally differentiated effector T cells capable of producing Th1- and Th17-associated cytokines as well as cytotoxic effector molecules (6). Although initially characterized for their antimicrobial functions, accumulating evidence indicates that MAIT cells can also directly target tumour cells and may exert either pro- or anti-tumour effects depending on contextual cues (7–9). However, within the tumour microenvironment, MAIT cells frequently acquire a dysfunctional or exhausted phenotype and may even contribute to tumour progression (10).

Clinically, circulating MAIT cells are reduced in colorectal and gastric cancer patients, likely due to tumour infiltration, where they display elevated expression of inhibitory receptors such as PD-1, Tim-3 and CD39 (2, 3, 11). In hepatocellular carcinoma (HCC), tumour-infiltrating MAIT cells express immune checkpoint molecules including PD-1 and CTLA-4, with conflicting associations to clinical outcome (4). Whereas higher MAIT cell infiltration has been found to correlate with poorer clinical outcomes, single-cell RNA sequencing analyses reveal that reduced MAIT cell abundance within tumours relative to healthy liver tissue correlates with a poor prognosis, suggesting an anti-tumour role for these cells (5). This dual role might be particularly plausible given that peripheral human MAIT cells express a variety of activating NK cell receptors, such as *Natural Killer Group 2 Member D* (NKG2D) (6). These receptors enable MAIT cells to mediate cytotoxic functions against MR1-negative tumours independently of their TCR (9). Notably, receptor expression varies by tissue, suggesting functional heterogeneity shaped by the microenvironment (7, 8). Despite this, NK cell receptor expression on tumour-resident MAIT cells remains poorly characterized. In this study, we conducted a comprehensive analysis of NK cell receptor expression on MAIT cells in peritumoral and tumour tissues from five GI cancer types. We compared these results with those from conventional T cells and NK cells, and further assessed the phenotypic and functional profiles of MAIT cells.

## Materials and methods

### Sex as a biological variable

All participants had a confirmed diagnosis of malignant disease, including both male and female patients.

### Patients

Macroscopic tumour specimens were obtained at surgical resection from patients with hepatocellular carcinoma (HCC, n = 30), colorectal liver metastases (CRLM, n = 15), colorectal cancer (CRC, n = 17), pancreatic ductal adenocarcinoma (PDAC, n = 17), and gastric cancer (GC, n = 18) (**Table 1**). Adjacent peritumoral tissue and paired peripheral blood samples were collected simultaneously. Patients had no concomitant inflammatory diseases and were not receiving immunosuppressive therapy. Peripheral blood from age-matched healthy donors without known systemic or inflammatory conditions served as controls (n=24). The primary endpoint was the frequency and functional profile of MAIT cells in tumour tissue. Variables included tissue compartment, tumour entity, and patient characteristics (e.g., age, sex). Potential confounders comprised inflammatory status, prior treatments, and metastatic disease. Longitudinal samples were available from seven patients. Clinicopathologic data were extracted from medical records. MAIT cell frequency, phenotype, and functional capacity were assessed by multiparameter flow cytometry using standardized antibody panels (**Supplementary Tables 1, 2**). Tumour diagnosis and organ origin were confirmed by histopathologic evaluation according to WHO criteria. All experimental procedures were performed using identical protocols to ensure comparability across tumour types and tissue compartments. The study was conducted in accordance with the Declaration of Helsinki and the Declaration of Istanbul and approved by the Ethics Committees of the Charité – Universitätsmedizin Berlin (EA4/128/16, EA1/353/16, EA4/065/20) and the Medical Faculty of Leipzig University (322/17-ek; 178/16-lk). Written informed consent was obtained from all participants; consent for this secondary analysis was waived where applicable.

**Table 1:** Patient demographics.

Variable	n (%)	Age min/max (years)	Age mean (years±SD)	Alcohol/Nicotin (%)	IMiDs (%)
Patients:					
Total number (%)	97 (100)				
Sex (male/female/ns)	61 (62.9)/ 36 (37.1)/ 0 (0.0)				
Cause of surgery:					
*Liver (n=45):*		50/87			11 (24.4)
HCC (%)	30 (66.7)		70.7±8.6	10 (33.3)/7 (23.3)	
Cause (MASH/MASLD/Viral) (%)	8 (26.6)/ 7 (23.3)/ 2 (6.7)				
Cause Alcohol/ns (%)	3 (10.0)/ 10 (33.3)				
AFP (>10ng/ml; n=20) (%)	8 (40.0)				
CRLM (%)	15 (33.3)	56/80	64.8±10.9	1 (6.7)/5 (33.3)	
CA19-9 (>27kU/l; n=4) (%)	2 (50.0)				
*Colon (n=17):*		23/85			1 (5.9)
CRC (%)	17 (100)		67.3±18.5	3 (17.6)/2 (11.8)	
CA19-9 (>27kU/l; n=4) (%)	0 (0.0)				
*Pancreas (n=17):*		41/84			4 (23.6)
PDAC (%)	17 (100)		64.7±12.1	3 (17.6)/6 (35.3)	
CA19-9 (>27kU/l; n=17) (%)	12 (70.6)				
*Stomach (n=18):*		39/83			13 (72.2)
GC (%)	18 (100)		65.8±11.7	3 (16.6)/5 (27.8)	
CA19-9 (>27kU/l; n=10) (%)	5 (50.0)				
CA72-4 (>6kU/l; n=7) (%)	2 (28.6)				
Tumour-related deaths (n=8)	8 (100)				
Healthy donors:					
Total number (%)	24 (100)	47/85	66.9±11.4	0 (0.0)/5 (20.8)	1 (4.2)
Sex (male/female/ns)	11 (45.8)/ 13 (54.2)/ 0 (0.0)				

Not all patients were included in all experiments due to limited access to tissue. ns: not specified. IMiDs, Immunomodulatory drugs; HCC, Hepatocellular Carcinoma; MASH, Metabolic Dysfunction-associated Steatohepatitis; MASLD, Metabolic dysfunction-associated steatotic liver disease; CRLM, Colorectal Liver Metastases; CRC, Colorectal Cancer; PDAC, Pancreatic Ductal Adenocarcinoma; GC, Gastric Cancer.

### Isolation of primary cells from human tissues

Tumour and matched peritumoral tissues from liver, pancreas, intestine, and stomach were mechanically dissociated into small fragments to generate single-cell suspensions. For liver and pancreatic samples, enzymatic digestion was performed in RPMI 1640 medium (Corning, REF: 10-040-CV) supplemented with 10% fetal calf serum (Gibco, REF: A5256801), 1% penicillin–streptomycin (Sigma-Aldrich, REF: P0781), collagenase II (1 mg/mL; Worthington, REF: LS004176), and DNase I (10 U/mL; Roche, REF: 11284932001). Intestinal and gastric tissues were digested under identical conditions except that collagenase IV (1 mg/mL; Worthington, REF: LS004188) was used instead of collagenase II. Samples were incubated for 45 minutes at 37 °C in a shaking water bath. Enzymatic activity was terminated by adding enzyme-free RPMI medium, and cell suspensions were filtered through a 100-µm cell strainer (VWR, REF: 732-2759). Cells were washed with PBS and centrifuged (10 minutes, 1500 rpm, 4°C). Mononuclear cells (MNCs) were isolated using Pancoll human (PAN-Biotech GmbH, REF: P14-60500; density 1.077 g/mL) by density gradient centrifugation (20 minutes, 2000 rpm, 1 deceleration/4 acceleration, room temperature). The interphase was collected, transferred to 50 mL Falcon tubes (Corning/Falcon, REF: 352070), washed with PBS supplemented with bovine serum albumin, and centrifuged (10 minutes, 1500 rpm, 4°C). Peripheral blood mononuclear cells (PBMCs) were isolated using the same Pancoll-based density gradient protocol. Cell numbers were determined using the CASY cell counting system (OLS, Germany; RRID: SCR_002080). Cells were either processed immediately for downstream analyses or cryopreserved in fetal bovine serum (FBS) supplemented with 10% dimethyl sulfoxide (Carl Roth, REF: A994.2).

### Flow cytometry and data acquisition

Typically, 1–2 × 10^6^ cells were stained for 20 minutes with fluorophore-conjugated antibodies (**Supplementary Table 1**). MAIT cells were identified as viable CD3^+^Vα7.2^+^CD161^+^CD8^+^ cells after exclusion of non-target populations using a dump channel containing Live/Dead, CD19, and CD14 to exclude dead cells, B cells, and myeloid cells, respectively, according to the gating strategy shown in **Supplementary Figure 1A**. For functional assays, cells were stained ex vivo with anti-CD69 antibody (BioLegend, REF: 310922) for 20 minutes at room temperature (RT) to enable quantification of activation prior to culture, as CD69 signal stability was maintained during subsequent incubation. Cells were then cultured in RPMI 1640 medium supplemented with 0.3 mg/mL glutamine, 100 U/mL penicillin, 0.1 mg/mL streptomycin, 10% human AB serum (Pan Biotech, REF: P30-2701), and recombinant human IL-15 (50 ng/mL; Miltenyi Biotec, REF: 130-093-955) or Phorbol-12-myristat-13-acetat (25 ng/ml; Sigma-Aldrich, REF: P8139)/Ionomycin calcium salt (1µg/ml; Sigma-Aldrich, REF: I0634) (PMA/Iono). To allow intracellular cytokine detection, brefeldin A (10 µg/mL; Cayman Chemical, REF: CAY11861-25) was added 4 hours before the end of the stimulation period (**Supplementary Figures 2A, B**). For analysis of XBP1s pathway activation, cells were stimulated with tunicamycin (1.25 µg/mL; Sigma-Aldrich, REF: T7765-1MG) or thapsigargin (1.25 µg/mL; Sigma-Aldrich, REF: T9033-5MG) for 24 or 96 hours. Unstimulated ex vivo cells served as controls in each experiment. Flow cytometric acquisition and analysis were performed using FlowJo v10 (BD; RRID: SCR_008520) and analyzed according to the gating strategy displayed in **Supplementary Figures 1**, **2**. Statistical analyses and graphical visualization were conducted using GraphPad Prism 8 (GraphPad, La Jolla, CA, USA; RRID: SCR_002798).

### Enrichment of MAIT and NK cells

MAIT cells were enriched from PBMCs by magnetic-activated cell sorting (MACS). Briefly, 4 × 10^7^ PBMCs were centrifuged and resuspended in 150 µL PBS supplemented with bovine serum albumin (PBS/BSA). Cells were incubated with 5 µL FcR Blocking Reagent (Miltenyi Biotec, REF: 130-059-901) and 2 µL Vα7.2-APC antibody (1:50; BioLegend, REF: 351708) for 15 minutes on ice. After washing with 4 mL PBS/BSA and centrifugation (300 × g, 6 minutes), cells were resuspended in 120 µL PBS/BSA and incubated with 30 µL APC-conjugated Nanobeads (Miltenyi Biotec, REF: 130-090-855) for 20 minutes at 4°C. Magnetic separation was performed using LS columns (Miltenyi Biotec, REF: 130-042-401) according to the manufacturer’s instructions. The positive fraction contained enriched MAIT cells, whereas the negative flow-through was retained for subsequent NK cell isolation. MAIT cell purity was assessed by flow cytometry; samples with <90% purity were subjected to a second magnetic separation. NK cells were isolated from the MAIT-depleted fraction by negative selection using the MojoSort™ Human NK Cell Isolation Kit (BioLegend, REF: 480053). Cells were centrifuged (1500 rpm, 6 minutes, 4°C), resuspended in 100 µL PBS/BSA, and incubated with 10 µL Biotin-Antibody Cocktail for 15 minutes on ice. After washing (300 × g, 6 minutes), cells were incubated with 10 µL Streptavidin Nanobeads for 15 minutes. The suspension was applied to LD columns (Miltenyi Biotec, REF: 130-042-901), and the negative fraction containing purified NK cells was collected. Purity was confirmed by flow cytometry after staining with CD56-BV785 (1:200; BioLegend, REF: 362550) and consistently exceeded 90%.

### Culture of the target cell line K562

The K562 cell line (chronic myelogenous leukaemia; ATCC CCL-243; RRID: CVCL_K562) was cultured in RPMI 1640 medium containing 0.3 mg/ml glutamine 100 U/ml penicillin, 0.1 mg/ml streptomycin and 10% FBS. Cells were split at a ratio of 1:10 every three to four days.

### Cytotoxicity assay

K562 target cells (ATCC CCL-243) were labeled with CellTrace Violet (CTV; Thermo Fisher Scientific/Invitrogen, REF: C34571) following the manufacturer’s instructions. Briefly, 1 × 10^7^ cells were centrifuged at 600 × g for 3 min and resuspended in 500 µl PBS. A staining solution was prepared by mixing 2.5 ml PBS with 1 µl CTV (1:2500), and 500 µl of the cell suspension was added. Cells were incubated for 4 min at room temperature in the dark, after which the reaction was quenched with 10 ml PBS/BSA. Labeled cells were centrifuged at 1500 rpm for 6 min at 4°C, resuspended in 5 ml PBS/BSA, and staining quality was confirmed by flow cytometry (BV421 channel). For ex vivo killing assays, freshly isolated, non-stimulated MAIT or NK cells were co-cultured with CTV-labeled K562 target cells. Effector and target cell numbers were determined using a CASY cell counter, and effector-to-target (E:T) ratios were adjusted accordingly. Where indicated, effector cells were pre-incubated with 2 µl antibody (20 µg/ml) for 15 min at 37°C before addition of target cells. Co-cultures were set up in 96-well U-bottom plates and incubated for 12 h at 37°C and 5% CO_2_. After incubation, 100 µl PBS/BSA containing 2 µl propidium iodide (PI; 1:10 pre-diluted; BD Biosciences, REF: 556463) was added per well, and target cell death was assessed by flow cytometry (PerCP-Cy5.5/PE channel). For IL-15-stimulated killing assays, MAIT or NK cells pretreated with IL-15 (50 ng/ml; Miltenyi Biotec, REF: 130-093-955) for 12–96 h were used as effectors. After stimulation, cells were washed twice with PBS/BSA, counted, and co-cultured with CTV-labeled K562 cells at defined E:T ratios in 96-well plates. If indicated, effector cells were pre-incubated with 2 µl inhibitory antibody (20 µg/ml) for 15 min at 37°C prior to addition of target cells. Co-cultures were incubated for 12 h at 37°C and 5% CO_2_, and cytotoxic activity was quantified by PI staining. Results were normalized to spontaneous target cell death in control wells (**Supplementary Figures 1B–D**).

### Analysis of single cell RNA sequencing data

The published human single-cell sequencing data were obtained through dbGaP (accession number: phs003279.v1.p1) as anndata files preprocessed according to the procedure described in Ruf et al. (10). For this study, only samples with the location “core” or “adjacent” were considered, while the region annotation “rim” was excluded. Furthermore, only MAIT identified via their TCR from bulk CD45^+^ cells were included in the analysis. No clustering beyond the identification as MAIT cell and the split between annotated regions was performed. For downstream analyses, scanpy version 1.12 was used in conjunction with anndata version 0.12.10. Samples were merged with anndata concat with the options merge=“none” and join=“inner”. As no clustering was performed, we did not run further integration steps. Plots were generated using scanpy.pl functionality.

### Statistics

The Kolmogorov-Smirnov test was used to assess data distribution, or the Shapiro-Wilk test if n was too small. Depending on normal distribution or not, 1-way ANOVA (with Holm-Sidak´s *post-hoc*), Kruskal-Wallis test (with Dunn *post-hoc*) or Friedman test were chosen for multiple comparisons. For two-group comparisons, unpaired t test (2-tailed) or Mann-Whitney test (2-tailed) were used. The relationship between two variables was examined by simple linear regression analysis. For analysis of contingency tables, *Fisher’s exact* test was applied. For OMIQ analysis flow-cytometry data from different patient cohorts were pooled together and separated into relevant experimental groups. The files were loaded into OMIQ (Dotmatics, USA) and clustered using either Flow Self-Organizing Map for cytometry data (FlowSOM; RRID: SCR_016899) or Graph-based clustering for single-cell data (PhenoGraph; RRID: SCR_016919) and visualized using optimized T-distributed stochastic neighbor embedding (opt-SNE). The clusters were overlaid in an opt-SNE plot. The overlaid clusters were either compared between experimental groups or checked for the expression levels of various markers using color gradient. In all tests, a value of p<0.05 was considered significant. Missing data were excluded from the relevant analyses. No imputation was performed.

## Results

### Compartment-specific distribution of MAIT cells across gastrointestinal tumour entities

We analysed the frequency of CD8^+^Vα7.2^+^CD161^+^ MAIT cells across five GI tumour entities (HCC, CRLM, CRC, PDAC, and GC) using paired peripheral blood, peritumoral, and tumour tissue samples from 97 patients (**Figure 1A**). In HCC and CRLM, MAIT cells were significantly enriched in peritumoral tissue compared with peripheral blood but were markedly reduced within tumours relative to matched peritumoral regions (**Figures 1B, C**). In contrast, MAIT cell frequencies were comparable across blood, peritumoral, and tumour tissues in CRC, PDAC, and GC, although CRC and PDAC displayed a trend towards reduced MAIT cell frequencies in peritumoral tissue compared with peripheral blood (**Figure 1C**). To determine whether these alterations were specific to MAIT cells, we analysed conventional CD8^+^ T cells and CD3^−^CD56^+^ NK cells from the same samples. Similar to MAIT cells, CD8^+^ T cells were enriched in peritumoral tissue but reduced within HCC and CRLM tumours. Higher frequencies of NK cells were identified in peripheral blood and peritumoral tissue from HCC and CRLM patients but were less frequent in CRC, PDAC, and GC. Their frequency was further reduced in CRLM tumours (**Figure 1C**). Given the larger HCC cohort and the reported age-associated decline in MAIT cells (12), we next examined the relationship between age and MAIT cell frequencies. While circulating MAIT cell frequencies declined with increasing age relative to age-matched healthy controls and peritumoral tissue, intratumoral MAIT cell frequencies remained unchanged (**Figures 1D, E**). No age-related associations were observed for CD8^+^ T cells, whereas circulating NK cell frequencies exhibited a similar age-associated decline in patients with HCC (**Supplementary Figures 3A, B**). Notably, intratumoral MAIT cell frequencies were observed in relation to patient survival (**Figure 1F**). Collectively, these findings reveal compartment- and tumour-specific alterations in MAIT cell distribution, characterized by preferential depletion of MAIT cells and cytotoxic CD8^+^ T cells in liver-associated tumours and distinct patterns of NK cell abundance across gastrointestinal cancers.

**Figure 1:**
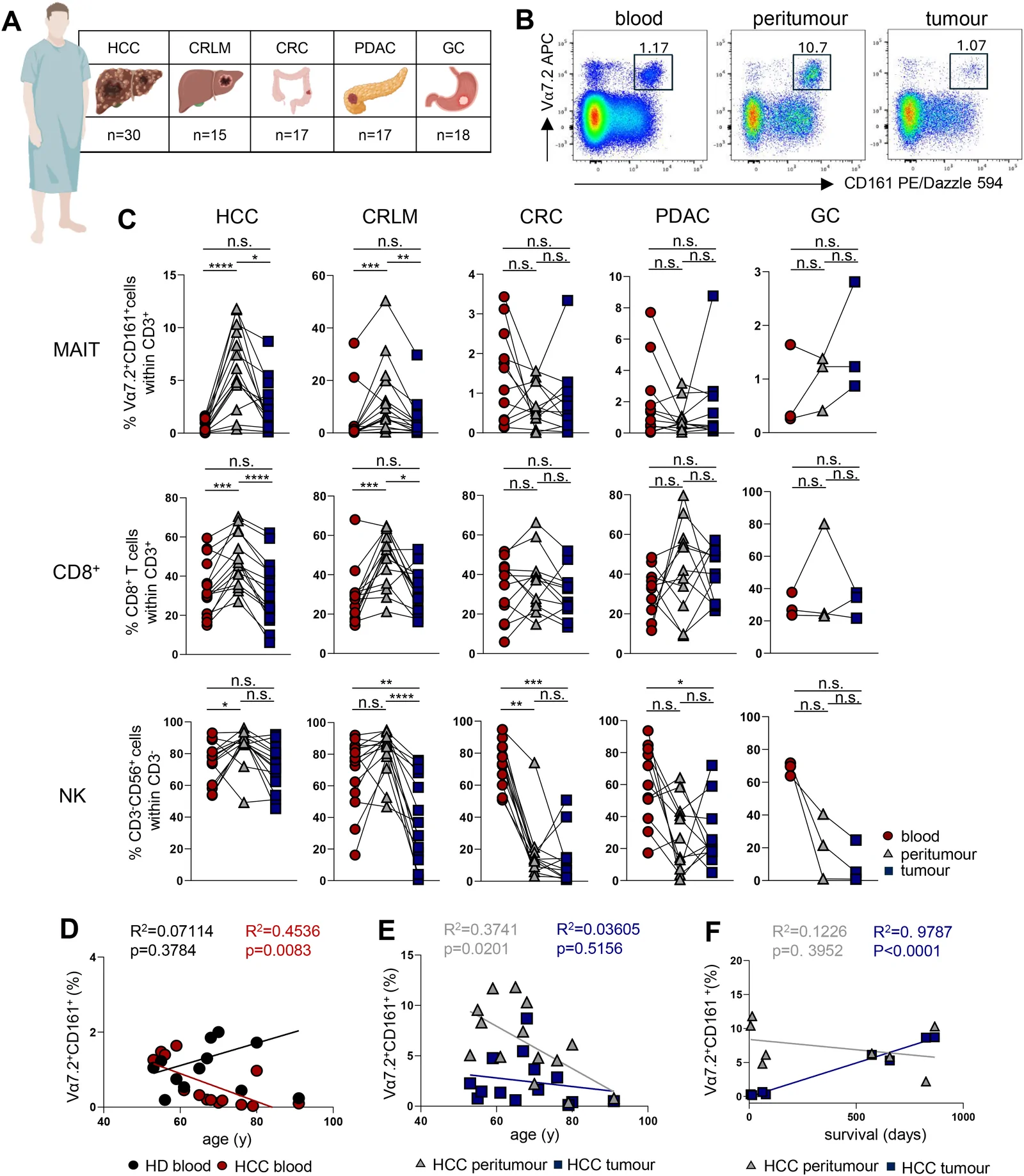
Frequencies of MAIT cells, CD8^+^ T and NK cells in five GI tumour types. **(A)** Summary of numbers for all GI tumour types included in the analysis. **(B)** Representative flow cytometry plots showing MAIT cells identified by flow cytometry in blood, peritumour and tumour tissue derived from a HCC patient. **(C)** Pairwise comparison of the indicated frequencies of MAIT cells, CD8^+^ T and NK cells derived from blood, peritumoral and tumour tissues. Only paired samples are shown. HCC: n = 14; CRLM: n = 15; CRC: n = 17; PDAC: n = 11; GC: n = 7. **(D)** Simple linear regression between peripheral MAIT cell frequencies derived from HCC patients and age-matched controls with chronological age. **(E)** Simple linear regression between HCC tissue-derived MAIT cell frequencies with chronological age. **(F)** Simple linear regression between peripheral MAIT cell frequencies derived from HCC peritumour and tumour tissue and patient survival (days). Non-significant (n.s.): p > 0.05; *p < 0.05; **p < 0.01; ***p < 0.001; ****p < 0.0001 as calculated by a two-tailed paired t-test or a two-tailed Wilcoxon matched-pairs signed-rank test (C).

### Tumour-derived MAIT cells display downregulated NK receptor expression

MAIT cells express several receptors that are also present on NK cells (6). We confirmed the expression of the NK-associated receptors NKG2D, CD56, FcγRIII (CD16), and the co-stimulatory molecule 2B4 (CD244) on MAIT cells isolated from matched peritumoral and tumour tissues across all GI tumour entities (**Figure 2A**). UMAP visualization demonstrated the distribution of MAIT cells across peripheral blood, peritumoral, and tumour compartments (**Figure 2B**), while opt-SNE analysis identified ten distinct MAIT cell clusters based on NK receptor expression patterns (**Figure 2C**). Notably, NKG2D expression was consistently reduced on tumour-derived MAIT cells across all tumour entities (**Figure 2D**), whereas its downregulation on conventional CD8^+^ T cells and NK cells was restricted to specific tumour types (**Supplementary Figures 4, 5**). Similarly, 2B4 expression was reduced on MAIT cells in all tumour entities except GC, with comparable reductions observed in CD8^+^ T cells and NK cells from HCC and CRC. In contrast, reduced CD16 expression was confined to MAIT cells from HCC and CRLM tumours, whereas CD56 expression was decreased in MAIT cells from HCC, CRLM, and CRC. A similar pattern was observed in CD8^+^ T cells for HCC, CRLM and GC but not in NK cells for the same entities (**Figure 2D**; **Supplementary Figure 4, 5**). Importantly, reduced NKG2D expression on tumour-derived MAIT cells correlated with markers of liver injury, including aspartate aminotransferase (AST) and alanine aminotransferase (ALT), in HCC (**Figures 2E, F**). Furthermore, NKG2D expression in both peritumoral and tumour tissues was associated with patient survival (**Figure 2G**). Collectively, these findings identify consistent downregulation of NKG2D as a defining feature of tumour-derived MAIT cells across GI cancers, accompanied by broader alterations in NK receptor expression.

**Figure 2:**
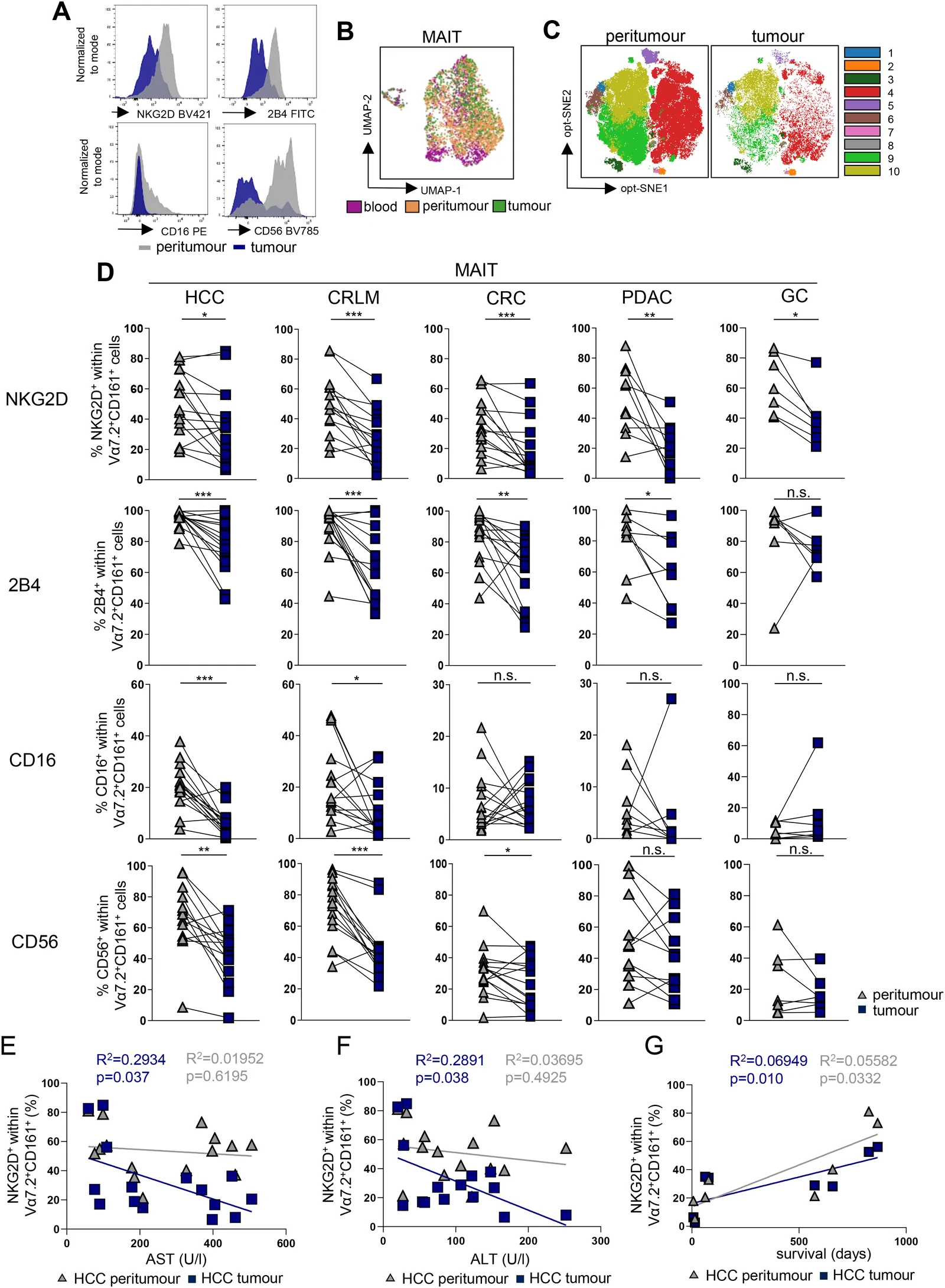
NK receptor expression profile of tissue-derived MAIT cells. **(A)** Representative histogram plots showing the expression of different NK cell receptors on peritumour- and tumour-derived MAIT cells from HCC. **(B)** Uniform Manifold Approximation and Projection (UMAP) was used to visualize the relative distribution of MAIT cells across blood, healthy and tumour tissue from HCC (n=13). Cells were down sampled to a total of 999,990 cells (333,333 from blood and healthy tissue, and 333,324 from tumour tissue). MAIT cells were identified by manual MAIT gating and UMAP analysis was performed using the following markers: Vα7.2, CD8, CD4, 2B4, CD16, CD161, CD3, NKG2D, and CD56. **(C)** Optimized t-distributed stochastic neighbour embedding (opt-SNE) plots illustrating clustering of MAIT cells according to their expression of NK cell markers, isolated from blood, peritumour and tumour samples derived from five GI cancer entities. A total of 233,241 cells were used for clustering from the pooled dataset using FlowSOM and the results were visualized using opt-SNE based on markers including Vα7.2, CD8, CD4, 2B4, CD16, CD161, CD3, NKG2D, and CD56. **(D)** The frequencies of NKG2D^+^, 2B4^+^, CD16^+^ and CD56^+^ MAIT cells derived from paired peritumour and tumour tissues from the following tumour entities: HCC: n = 14; CRLM: n = 15; CRC: n = 17; PDAC: n = 11; GC: n = 7. **(E, F)** Simple linear regression analysis between NKG2D^+^ MAIT cell frequencies isolated from HCC peritumour and tumour tissue and functional liver parameters (left panel: aspartate-aminotransferase, AST; right panel: alanine-aminotransferase, ALT). **(G)** Simple linear regression analysis between NKG2D^+^ MAIT cell frequencies isolated from HCC peritumour and tumour tissue and patient survival (days). Non-significant (n.s.): p > 0.05; *p < 0.05; **p < 0.01; ***p < 0.001, as calculated by a two-tailed paired t-test or a two-tailed Wilcoxon matched-pairs signed-rank test (D).

### Peripheral blood MAIT cells exhibit downregulated NKG2D across GI tumours

Across all cohorts, circulating frequencies of MAIT cells, CD8^+^ T cells, and NK cells were largely comparable between healthy donors and patients with the investigated cancer entities, with the sole exception of increased CD8^+^ T cell frequencies in patients with HCC and decreased NK cells in patients with PDAC (**Supplementary Figure 6**). In contrast, expression of the activating NK receptor NKG2D on peripheral blood lymphocytes varied markedly across tumour entities. NKG2D expression was significantly reduced on circulating MAIT cells from patients with HCC, CRLM, and CRC, with a similar reduction observed in CD8^+^ T cells. In NK cells, reduced NKG2D expression was observed in patients with HCC and CRC (**Figure 3A**). To determine whether these alterations were reversible following tumour removal, a subset of patients with HCC, CRLM, and CRC was longitudinally analysed for up to six months after surgical resection. While circulating lymphocyte subset frequencies remained stable during follow-up (**Figure 3B**), NKG2D expression significantly increased six months after tumour resection on MAIT cells, CD8^+^ T cells, and NK cells across all analysed cohorts (**Figures 3C–E**). Furthermore, NKG2D expression on peripheral MAIT cells was associated with overall survival (**Figure 3F**). Collectively, these findings demonstrate that reduced NKG2D expression on circulating MAIT cells in patients with HCC, CRLM, and CRC is reversible following tumour resection and is associated with clinical outcome.

**Figure 3:**
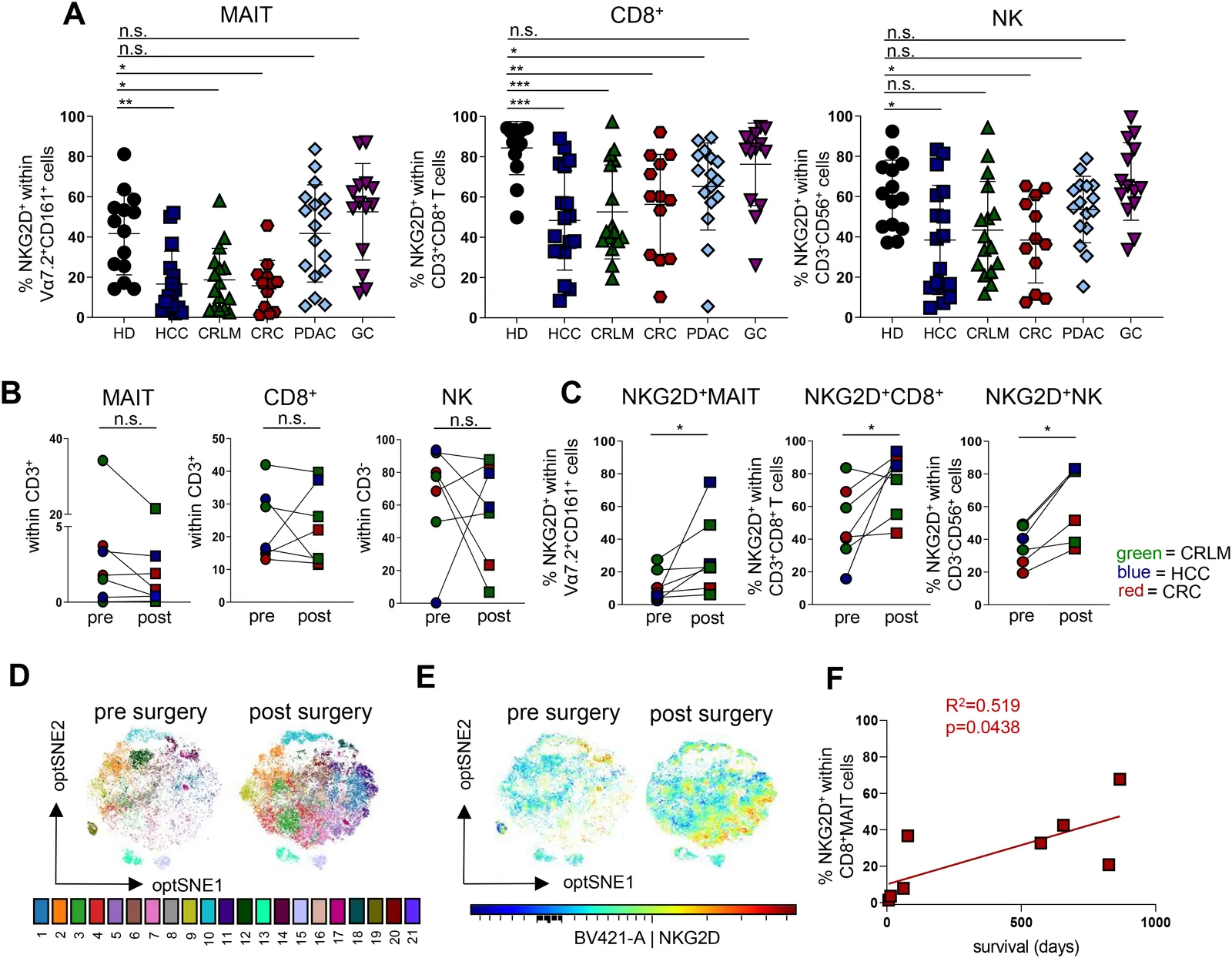
Expression of NKG2D on peripheral MAIT cells from HCC patients. **(A)** Expression of NKG2D on blood-derived MAIT cells, as well as on CD8^+^ T cells and NK cells, within different tumour entities, HD: n = 14; HCC: n = 18; CRLM: n = 16; CRC: n = 14; PDAC: n = 17; GC: n = 14. **(B)** Paired analysis of MAIT, CD8^+^, and NK cell frequencies in blood samples collected before surgical resection **(pre)** and ~6 months after surgery (post), HCC: n = 2; CRLM: n = 4; CRC: n = 2. **(C)** Paired analysis of NKG2D^+^ MAIT, CD8^+^ T, and NK cells within blood samples collected prior to surgical resection (pre) and ~6 months after surgery (post), HCC, n = 2; CRLM, n = 4; CRC, n = 2. **(D)** MAIT cells from patients pre- and post-surgery were clustered using Phenograph and visualized using opt-SNE to reveal the differential distribution of MAIT cells across these two groups in clustering and opt-SNE space (n=9 patients each pre- and post-surgery). **(E)** opt-SNE visualization of NKG2D expression pre- and post-surgery for peripheral MAIT cells. Cells were gated on live (Dump- Dump^-^CD3^+^CD161^+^Vα7.2^+^CD8^+^ cells. **(F)** Simple linear regression between NKG2D^+^ peripheral MAIT cell frequencies derived from HCC patients and survival (days). Non-significant (n.s.): p > 0.05; *p < 0.05; **p < 0.01; ***p < 0.001, as calculated by ordinary one-way ANOVA, Kruskal–Wallis test (3A), two-tailed paired t-test, two-tailed Wilcoxon matched-pairs signed-rank test (B, C) or simple linear regression (F).

### HCC-derived MAIT cells exhibit impaired effector function despite preserved tissue residency

Due to limited cell availability, subsequent functional analyses were performed on MAIT cells derived from HCC patients. We assessed the cytokine profile of circulating, peritumoral, and tumour-derived MAIT cells to determine whether their effector function was altered within the tumour microenvironment. MAIT cells from peritumoral tissue exhibited increased production of several effector molecules compared with peripheral blood-derived MAIT cells, with the exception of Granzyme B. In contrast, tumour-derived MAIT cells displayed significantly reduced IFNγ production, whereas TNFα and IL-2 showed a similar but non-significant trend towards decreased expression. IL-17 and Granzyme B production remained unchanged (**Figure 4A**). Consistent with these findings, tumour-derived CD8^+^ T cells showed significantly reduced production of IFNγ, IL-2, and TNFα. NK cells exhibited a comparable functional impairment, with reduced Granzyme B expression in tumour tissue compared with peripheral blood (**Figure 4A**). Furthermore, HCC-derived MAIT cells displayed reduced polyfunctionality, with fewer cells simultaneously producing three effector cytokines compared with their peritumoral counterparts, a pattern similarly observed in CD8^+^ T cells (**Supplementary Figure 7**). Circulating MAIT cells from patients with HCC exhibited a distinct functional profile compared with healthy controls, characterized by increased Granzyme B production and reduced TNFα and IL-17 expression, while IFNγ and IL-2 remained unchanged. Similar alterations were observed in conventional CD8^+^ T cells, whereas NK cells demonstrated selective enhancement of Granzyme B expression (**Supplementary Figure 8**). In summary, these findings demonstrate that peritumour-derived MAIT cells exhibit enhanced cytokine polyfunctionality, while tumour-derived MAIT cells undergo functional reprogramming with impaired effector capacity within the tumour microenvironment.

**Figure 4:**
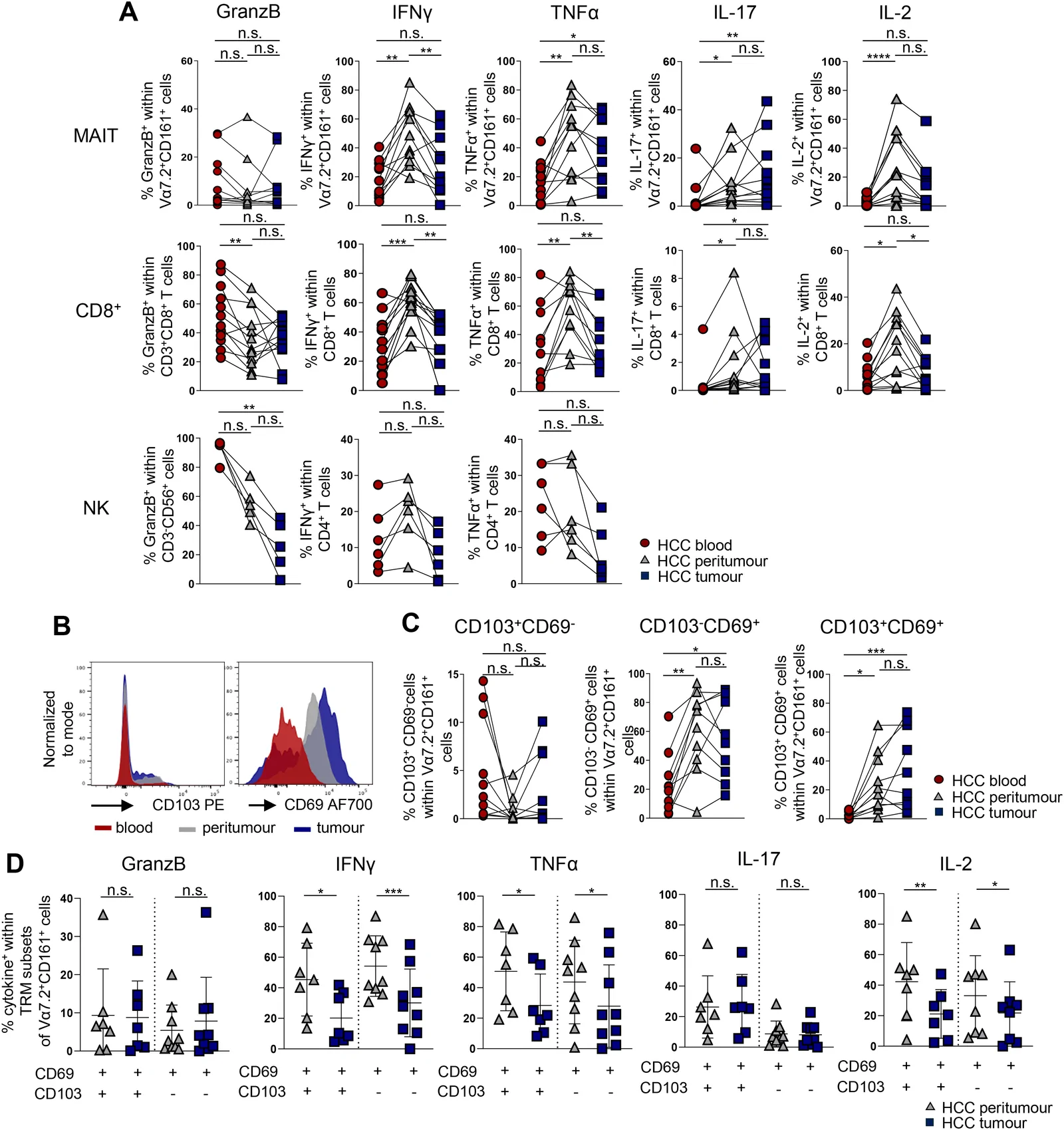
Effector function of MAIT cells, CD8^+^ T, and NK cells and tissue retention signatures of MAIT cells in HCC. **(A)** Cytokine frequencies of investigated lymphocyte subsets derived from peripheral blood, peritumoral and tumour tissue from HCC patients, n = 11–12/tissue after PMA/Iono stimulation for 6h. **(B)** Representative histogram plot of the tissue-resident molecules CD69 and CD103 expressed on MAIT cells from peripheral blood, peritumoral and tumour tissue. **(C)** Paired analysis of HCC-derived MAIT cells isolated from peripheral blood, peritumoral tissue, and tumour tissue, assessing the expression of tissue-residency-associated markers, n = 10. **(D)** Cytokine frequencies of tissue-resident MAIT cells, defined by the presence of CD103 and CD69, derived from HCC peritumour and tumour tissues after PMA/Iono stimulation for 6h. HCC, n = 7–9/tissue. Non-significant (n.s.): p > 0.05; *p < 0.05; **p < 0.01; ***p < 0.001, as calculated by ordinary one-way ANOVA, Kruskal–Wallis test (A, C), a two-tailed paired t-test or a two-tailed Wilcoxon matched-pairs signed-rank test (D).

We recently demonstrated that classical markers of tissue residency, including the integrin αE (CD103) and the type II C-type lectin receptor CD69, are expressed on MAIT cells in human renal tissue (13). Consistent with these findings, we detected CD103 and CD69 expression on MAIT cells isolated from HCC-derived peritumoural and tumour tissues (**Figures 4B, C**). However, the frequencies of CD103^+^, CD69^+^ single-positive, and CD103^+^CD69^+^ double-positive MAIT cell subsets did not differ significantly between tumour and peritumoural samples (**Figure 4C**). Interestingly, tumour-derived MAIT cells expressing CD69 alone or co-expressing CD103 and CD69 exhibited significantly reduced production of IFNγ, TNFα, and IL-2 compared with their peritumoural counterparts (**Figure 4D**). In contrast, Granzyme B and IL-17 production remained unchanged. These findings indicate that tumour-associated MAIT cells retain a tissue-resident phenotype while displaying impaired effector cytokine production within the tumour microenvironment.

### Cytotoxic capacity of HCC tissue-derived MAIT cells

Previous studies have shown that peripheral blood-derived MAIT cells can kill tumour cells (9, 14). In line with these findings, we evaluated the cytotoxic potential of MAIT cells, alongside NK cells, isolated from the peripheral blood of healthy donors and HCC patients. The cells were either stimulated with IL-15 for 96 hours or left untreated, after which their killing capacity was assessed against the tumour cell line K562 (**Figures 5A, B**). Both peripheral MAIT and NK cells demonstrated a significant increase in cytotoxicity following IL-15 activation. However, while the killing capacity of MAIT cells after stimulation was comparable between healthy donors and HCC patients, the killing ability of NK cells from tumour patients was significantly reduced compared with healthy controls (**Figures 5C, D**). Strikingly, we tested the killing capacity of liver-derived MAIT cells and observed a significant enhancement in the killing capacity triggered by IL-15 stimulation in peritumoral MAIT cells, but importantly, not in tumour-derived MAIT cells. In contrast, elevated cytotoxicity was displayed by NK cells from both peritumoral and tumour tissues upon IL-15 activation, with no significant difference between the two tissue types (**Figures 5E, F**).

**Figure 5:**
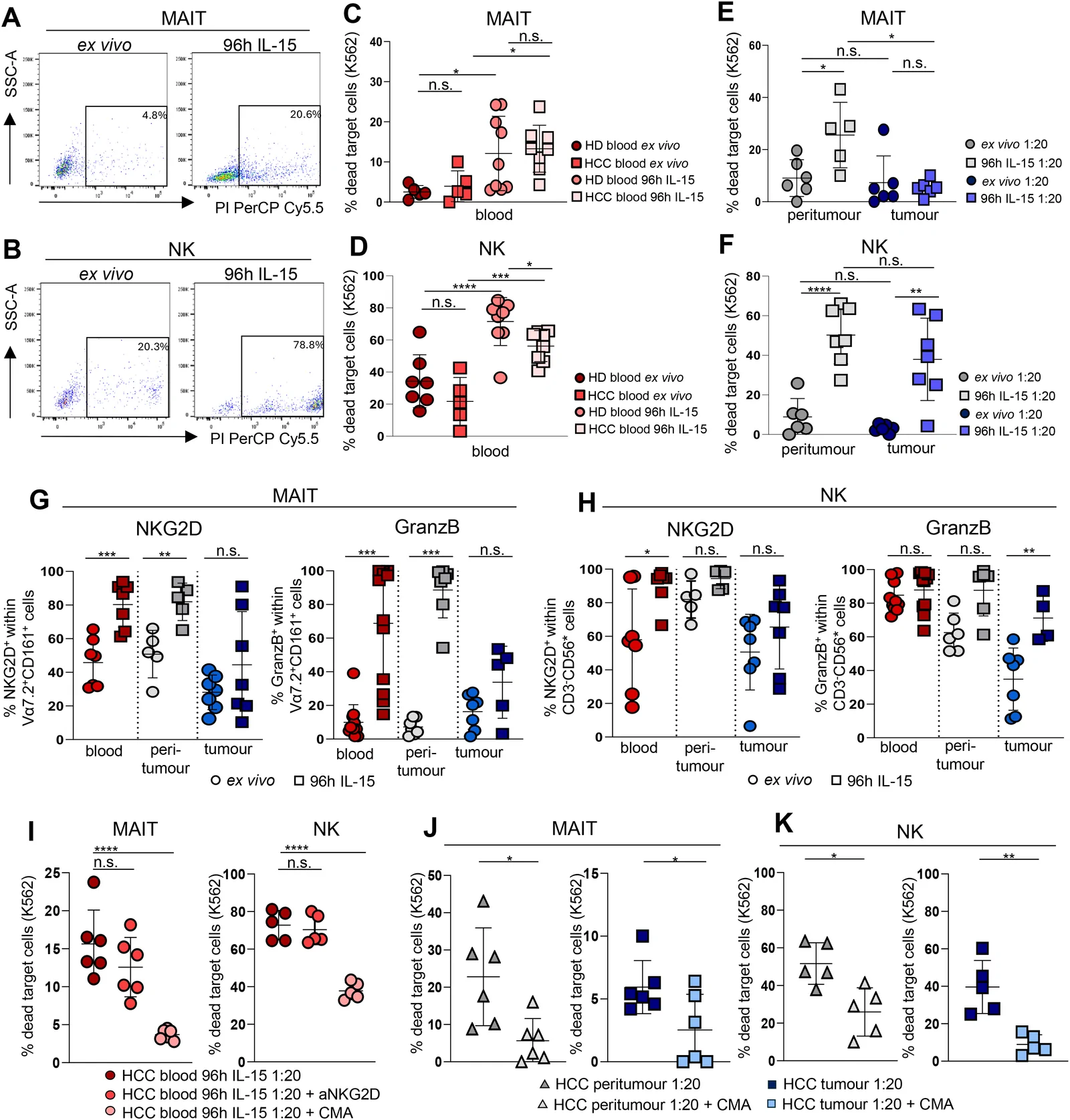
Cytotoxic capacity of HCC-derived MAIT cells. **(A, B)** Representative flow cytometry plots illustrating the cytotoxic potential of MAIT and NK cells ex vivo and following 96 hours of IL-15 stimulation. **(C, D)** Differences in cytotoxicity of blood-derived MAIT and NK cells from healthy participants (HD) and HCC patients (HCC), analyzed ex vivo and after IL-15 stimulation at an effector-to-target ratio of 20:1, n =7-8/group. **(E, F)** Differences in cytotoxicity of peritumour and tumour-derived MAIT and NK cells from HCC patients. The cytotoxicity assay was conducted either ex vivo or over a 96-hour period with IL-15 at a ratio of 1:20, n =5-7/group. **(G, H)** Induction of NKG2D and Granzyme B on peripheral blood, peritumour and tumour-derived MAIT cells after 96 hours of IL-15 stimulation. **(I)** Inhibition of the cytotoxic capacity of peripheral MAIT and NK cells from HCC patients following 96-hour IL-15 stimulation, using a neutralizing antibody against NKG2D or the Granzyme B inhibitor concanamycin A (CMA), at an ratio of 1:20, n = 5-6/group. **(J, K)** Inhibition of MAIT and NK cell cytotoxicity from HCC peritumoral and tumour samples using CMA after 96-hour IL-15 stimulation at an ratio of 1:20 (n = 5–6 per group). HD: n = 6–9; HCC: n = 5–6. Non-significant (n.s.): p > 0.05; *p < 0.05; **p < 0.01; ***p < 0.001, as calculated using ordinary one-way ANOVA, Kruskal–Wallis test (C–F, I), a two-tailed paired t-test or a two-tailed Wilcoxon matched-pairs signed-rank test (G, H).

As NKG2D was significantly downregulated on MAIT cells derived from tumours compared to peritumoral tissues from all entities investigated, we aimed to understand whether MAIT cell activation leads to NKG2D induction. Prolonged IL-15 stimulation (96 hours) enhanced NKG2D expression and Granzyme B production in peripheral blood and peritumoral MAIT cells from HCC patients; however, this inducible effector response was absent in tumour-derived MAIT cells (**Figure 5G**). In contrast to peripheral blood-derived NK cells, which upregulated NKG2D upon stimulation, NK cells isolated from tumour and peritumoral tissues failed to induce NKG2D expression. Nevertheless, tumour-derived NK cells retained the capacity to produce Granzyme B (**Figure 5H**). Thus, in parallel with NK cells, HCC tumour-derived MAIT cells exhibit a marked impairment in cytotoxic capacity that is not restored by IL-15 stimulation.

Given that both NKG2D and Granzyme B were induced following sustained IL-15 stimulation in peripheral and peritumour-derived MAIT cells but not in tumour-derived MAIT cells, we next examined whether the cytotoxic capacity of MAIT cells was dependent on these pathways by utilizing NKG2D blockade or pharmacological inhibition of Granzyme B. In contrast to recent reports (9), NKG2D blockade using a neutralizing antibody did not impair killing capacity in either peripheral MAIT or NK cells; however, inhibition of Granzyme B-mediated cytotoxicity with Concanamycin A (CMA) significantly reduced MAIT cell cytotoxicity (**Figure 5I**). This effect was observed in peritumoral and tumour-derived MAIT cells from patients with HCC (**Figure 5J**) and was similarly evident in NK cells (**Figure 5K**). Taken together, these data show that HCC tumour-derived MAIT cells exhibit impaired IL-15-induced cytotoxicity, accompanied by a failure to upregulate NKG2D and Granzyme B. Furthermore, MAIT cell-mediated killing was primarily dependent on Granzyme B rather than NKG2D signaling, highlighting a profound dysfunction of the cytotoxic programme within the tumour microenvironment.

### The ER stress–response factor XBP1s is inducible in MAIT cells

Spliced X-box binding protein 1 (XBP1s) is a key transcription factor acting downstream of IL-15 and AKT signaling that regulates cellular responses to endoplasmic reticulum (ER) stress. XBP1s binds to and recruits the transcription factor T-BET to the Granzyme B promoter, thereby enhancing its transcription. Through this mechanism, XBP1s promotes NK cell cytolytic activity against tumour cells and is essential for IL-15–mediated NK cell survival via anti-apoptotic pathways (15, 16). Here, we demonstrate the expression of the endoplasmic reticulum stress-responsive transcription factor XBP1s in both peripheral blood-derived and HCC tissue-resident MAIT cells, revealing a previously unrecognized feature of MAIT cell biology (**Figure 6A**). While XBP1s expression in peripheral MAIT cells did not differ between healthy donors and patients with HCC, it was reduced in peritumoral MAIT cells and further downregulated in tumour-derived MAIT cells (**Figure 6B**). Notably, peripheral MAIT cells from healthy donors exhibited higher basal XBP1s expression than CD8^+^ T or NK cells, whereas peripheral NK cells from tumour patients displayed the highest XBP1s expression overall (**Supplementary Figures 9A, B**).

**Figure 6:**
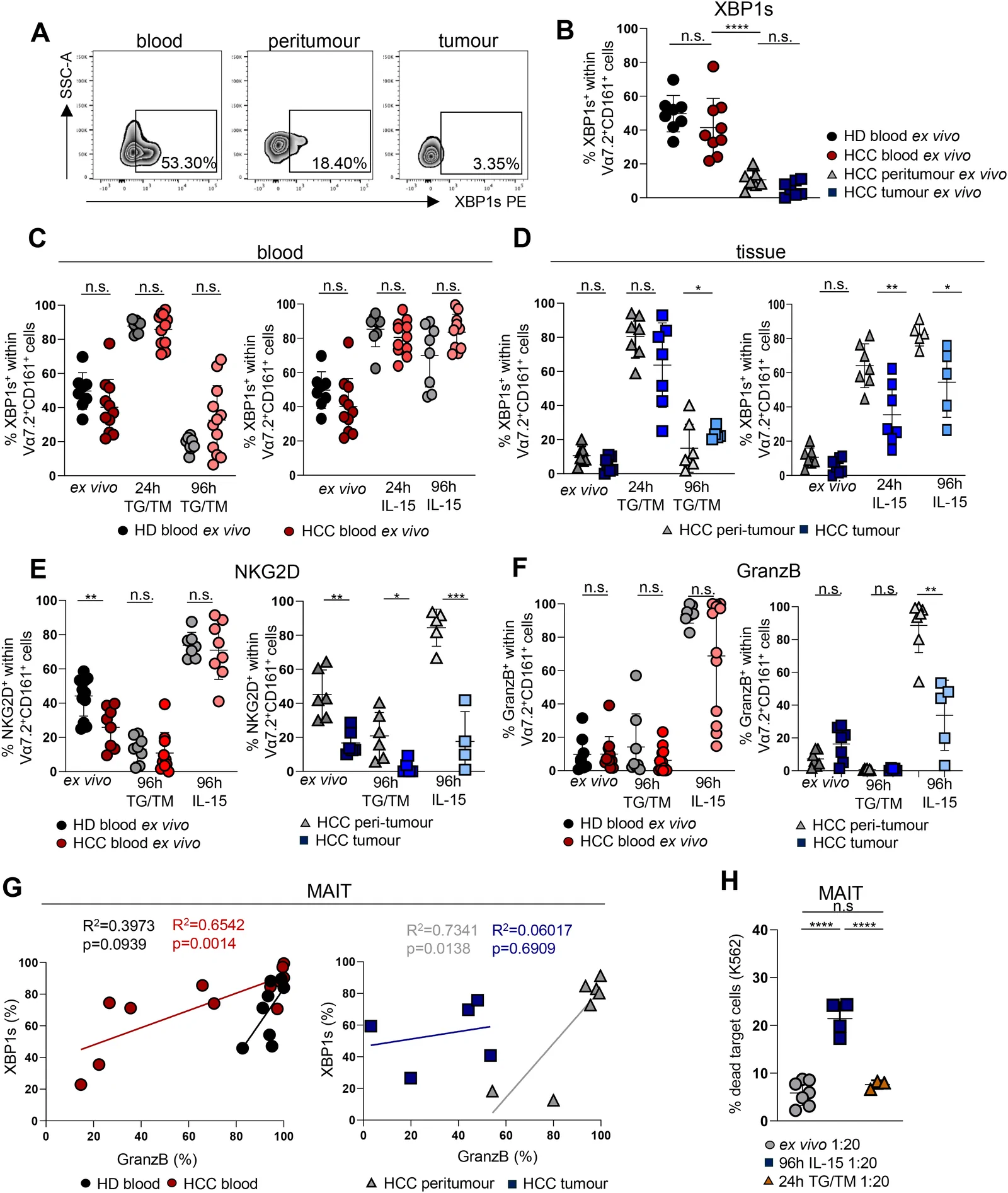
XBP1s activation in peripheral and tissue-derived MAIT cells. **(A)** Representative flow cytometry contour plots illustrating XBP1s expression in MAIT cells isolated from peripheral blood, peritumoral tissue, and tumour tissue of patients with HCC. **(B)** Frequencies of XBP1s^+^ MAIT cells in peripheral blood from age-matched healthy donors (HD, n = 8) and in peripheral blood- and tissue-derived (peritumoral and tumour) MAIT cells from patients with HCC, n=6-10/tissue. **(C)** Frequencies of peripheral XBP1s^+^ MAIT cells - isolated from healthy donors (HD, n = 8) and patients with HCC (n = 6–8), or from **(D)** peritumour and tumour HCC tissue - analyzed ex vivo or following functional stimulation to modulate cytotoxic capacity using the ER stress inducers thapsigargin (TG) and tunicamycin (TM), or IL-15 for 24 or 96 hours; n = 8–12/group. **(E)** Frequencies of peripheral MAIT cells expressing NKG2D or Granzyme B - isolated from healthy donors (HD, n = 8) and patients with HCC (n = 6–8), or from **(F)** peritumour and tumour HCC tissue - analyzed *ex vivo* or following functional stimulation to modulate cytotoxic capacity using the ER stress inducers TG and TM or IL-15 for 24 or 96 hours; n = 8–12/group. **(G)** Correlation analysis between XBP1s and Granzyme B in peripheral MAIT cells from age-matched healthy donors and HCC patients (left panel), and in peritumoral and tumour MAIT cells from HCC tissue (right panel) after IL-15 stimulation for 96 hours. **(H)** Cytotoxic capacity of peripheral MAIT cells isolated from healthy donors *ex vivo* or following 96-hour stimulation with IL-15 or the ER stress inducers TG and TM (n = 3–7 per group). Non-significant (n.s.): p > 0.05; *p < 0.05; **p < 0.01; ***p < 0.001, as calculated by a two-tailed paired t-test, two-tailed Wilcoxon matched-pairs signed-rank test (C–F), ordinary one-way ANOVA or Kruskal–Wallis test (B, H) and simple linear regression.

In peripheral MAIT cells from both healthy donors and patients with HCC, XBP1s expression was induced by the ER stressors thapsigargin (TG) and tunicamycin (TM), as well as by IL-15 (**Figure 6C**). However, XBP1s induction following TG/TM stimulation was transient and no longer detectable after 96 hours, whereas IL-15-mediated induction persisted throughout the 96-hour observation period. No differences in XBP1s expression were observed between peripheral MAIT cells from healthy donors and patients with HCC (**Figure 6C**). Consistent with these findings, XBP1s induction was also observed in tissue-derived MAIT cells following stimulation with TG/TM, or IL-15. However, IL-15-induced XBP1s expression was significantly lower in tumour-infiltrating MAIT cells compared with peritumoral MAIT cells. In contrast, ER stress-induced XBP1s activation was transient and, as observed in peripheral MAIT cells, was no longer detectable after 96 hours (**Figure 6D**). Similar patterns were observed in CD8^+^ T cells and NK cells (**Supplementary Figures 9A, B**).

Although treatment with TG/TM successfully induced expression of XBP1s, this alone was not sufficient to induce the NK-cell activation markers NKG2D or Granzyme B. In contrast, both NKG2D and Granzyme B were induced in response to IL-15 stimulation in peripheral blood and peritumoral tissue (**Figures 6E, F**). The extent of IL-15-induced Granzyme B expression positively correlated with XBP1s levels in MAIT cells derived from peripheral blood and peritumoral tissue, whereas this correlation was absent in tumour-derived MAIT cells (**Figure 6G**). While IL-15 stimulation enhanced MAIT-cell cytotoxicity, ER stress alone had no effect on the cytotoxic capacity of peripheral MAIT cells from healthy donors (**Figure 6H**). In summary, these data identify impaired XBP1s induction as a defining feature of HCC tumour-derived MAIT cells and reveal the IL-15–AKT–XBP1s signaling axis as a critical driver of Granzyme B production and cytotoxic effector function.

Finally, to validate our findings, we re-analyzed previously published scRNA sequencing data of CD45^+^ immune cells isolated from six matched HCC tumours and adjacent liver tissue (10). Within the MAIT cell compartment, we observed reduced expression of *KLRK1* (encoding NKG2D), *NCAM1* (CD56), CD69 and XBP1 in tumour-derived cells compared with peritumoral counterparts, corroborating our surface protein data. In addition, transcripts associated with effector function and cellular activation, including IFNG and XBP1, were likewise diminished (**Figure 7**).

**Figure 7:**
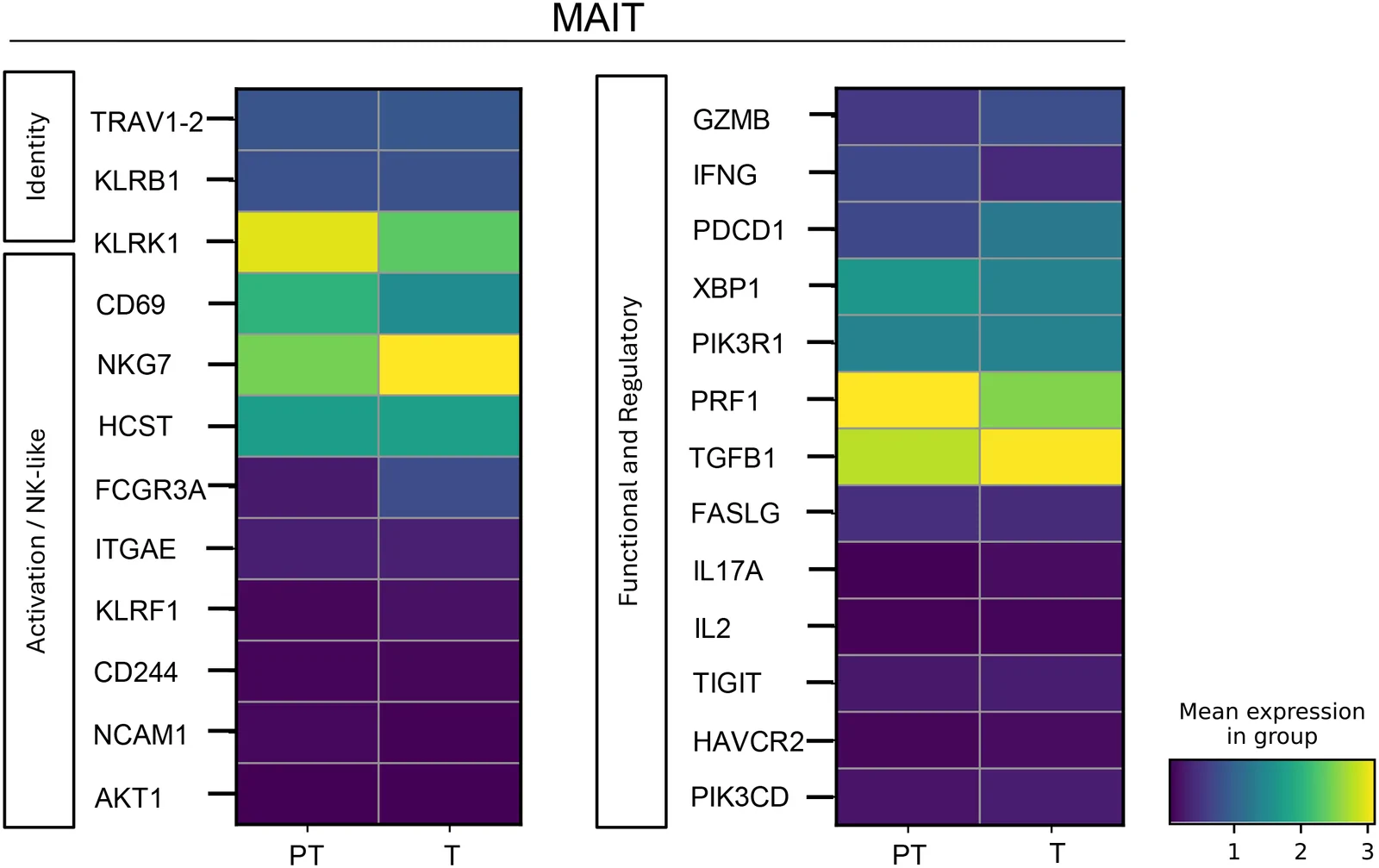
Transcriptomic confirmation of flow cytometric data. Matrix plots showing expression of selected markers of identity, activation, functionality and regulation in CD8^+^MAIT cells derived from the single-cell sequencing dataset Ruf et al. (10). PT, peritumour; T, tumour.

## Discussion

Although MAIT cells have been implicated in tumour immunity through their cytotoxic and cytokine-secreting functions, their precise role in cancer remains incompletely defined (3, 12, 17), and reported frequencies vary considerably across tumour types. Such variability is likely driven by substantial methodological heterogeneity, which limits direct comparison between studies. Moreover, interactions between MAIT cells and other immune cell types, including macrophages (18) and mast cells (19), have been shown to influence MAIT cell phenotype and function. Conversely, MAIT cells can enhance the activity of NK cells isolated from patient tumour samples (17).

Previous reports have described increased MAIT cell numbers in colonic tissue from patients with CRC, alongside reduced peripheral MAIT cell frequencies in CRC or GC (2, 3, 20–22). In contrast, our findings indicate comparable or even increased peripheral MAIT cell frequencies in CRC and PDAC. This discrepancy may reflect differences in our cohort composition, including the relatively small number of patients with these tumour entities. Data on MAIT cells in PDAC remain sparse; however, our observations suggest a broadly similar distribution of MAIT cells across blood, peritumoral tissue and tumour tissue, arguing against pronounced compartmentalization in this tumour entity.

Consistent with previous reports, we observed a pronounced enrichment of MAIT cells in the liver, with the highest frequencies detected in CRLM, surpassing those observed in HCC (12). These findings suggest tumour-specific and/or microenvironment-driven differences in the recruitment or retention of MAIT cells within the hepatic niche. While an age-associated decline in MAIT cell frequencies has been proposed (12), this relationship was evident in our HCC cohort but not in age-matched controls, implying that age-related effects may be context-dependent and influenced by the underlying disease. Notably, elevated intra-tumoral MAIT cell frequencies were observed in relation to patient survival. Furthermore, liver-resident MAIT cells exhibited a tissue-resident signature comparable to that described in lung cancer as well as in intestinal and renal tissues (10, 20, 21). In agreement with previous studies (4, 19), ex vivo-isolated MAIT cells from blood and liver tissues showed low basal Granzyme B expression that was strongly induced by IL-15 but significantly blunted in tumour patients. Consistent with reports in CRC and CRLM, liver-derived MAIT cells were more polyfunctional than blood-derived cells; however, this capacity was reduced in tumour-derived MAIT cells (4, 20, 21, 23).

The expression of NK cell receptors on MAIT cells is well established. For instance, NKG2D is readily detectable on liver-resident MAIT cells from healthy tissue and on MAIT cells within lung tumours (8, 24). In contrast, we observed a pronounced reduction of NKG2D on tumour-resident MAIT cells across all tumour types examined, accompanied by decreased expression of 2B4, a key regulator of NK cell activation (25). Notably, re-expression following tumour resection occurred not only in peripheral MAIT cells but also in CD8^+^ T cells and NK cells, suggesting a systemic effect of the disease. Activated MAIT cells from human liver perfusates have been shown to mediate NKG2D-dependent, Granzyme B-driven cytotoxicity independently of TCR/MR1 signaling (9). While inhibition of Granzyme B significantly reduced K562 cell killing, blockade of FasL or TRAIL was ineffective (9), contrasting with more recent data indicating a requirement for FasL, together with MAIT cell-derived IFNγ and TNFα, to prevent peripheral MAIT cell-mediated killing of HCC cells (14). Consistent with these findings, we confirmed the cytotoxic capacity of both blood- and liver-derived MAIT cells. This cytotoxicity could be induced by IL-15 in peripheral and peritumoral MAIT cells from HCC patients, but not in tumour-derived MAIT cells. Blocking NKG2D did not affect cytotoxicity, suggesting that this receptor does not play a major role in mediating target cell killing. Nevertheless, in line with previous observations in healthy liver-derived MAIT cells (8), inhibition of Granzyme B markedly reduced the cytotoxic capacity of activated MAIT cells from peripheral blood, peritumoral tissue, and tumour tissue of HCC patients. Given our observation that MAIT cells, albeit to a lesser extent than NK cells, are capable of killing tumour cells after stimulation with IL-15, and that IL-15 is a key regulator of NK cell cytotoxicity (16), we investigated whether IL-15 similarly induces activation of XBP1s (spliced form of XBP1) in MAIT cells, a transcription factor known to regulate NK cell function. XBP1 activation has previously been reported in dendritic cells and T cells within ovarian cancer microenvironments, where targeting XBP1 in either cell type restored anti-tumour immunity and consequently improved host survival (24, 26). Moreover, XBP1 has been shown to regulate the cytokine transcriptional program in macrophages, thereby promoting cancer progression (18). We observed a pronounced upregulation of XBP1s in peripheral blood-derived MAIT cells, with expression levels exceeding those observed in conventional T cells, whereas HCC tissue-derived MAIT cells exhibited comparatively lower XBP1s expression. This pattern contrasts with findings in breast and ovarian cancer, where XBP1 upregulation in tumour-infiltrating T cells was associated with reduced T cell infiltration and diminished IFNγ mRNA expression (24). Notably, XBP1s expression in MAIT cells derived from HCC was inducible by IL-15 and ER stress activators in peripheral blood- and peritumoral-derived cells; however, this induction was markedly attenuated in tumour-derived MAIT cells following IL-15 stimulation, reflecting their overall functional impairment. Granzyme B expression was induced exclusively by IL-15 and correlated with XBP1s expression in peripheral blood MAIT cells; moreover, inhibition of Granzyme B resulted in a marked reduction of MAIT cell cytotoxicity in patients with HCC.

In summary, our findings provide a comprehensive map of MAIT cell distribution across five gastrointestinal cancers and highlight downregulation of NK cell receptors, with NKG2D emerging as a candidate marker for tumour presence and patient survival. Importantly, we reveal a profound loss of MAIT cell polyfunctionality and Granzyme B-mediated cytotoxicity in HCC, which cannot be restored by IL-15 stimulation or XBP1s pathway activation. However, further studies are necessary to clarify the underlying molecular mechanisms driving this dysfunctional MAIT cell phenotype and to identify the upstream factors responsible for its development within the tumour microenvironment. In addition, future studies with larger patient cohorts will be required to account for patient heterogeneity and determine whether specific clinical characteristics are associated with the extent of MAIT cell dysfunction.

## Data Availability

The datasets presented in this study can be found in online repositories. The names of the repository/repositories and accession number(s) can be found below: https://www.ncbi.nlm.nih.gov/gap/, phs003279.v1.p1.
