# Supplementary material for: Comparative profiling of MAIT cells across gastrointestinal cancers identifies conserved NK receptor dysregulation and HCC-specific defects in cytotoxicity and XBP1s signaling
Source: Front Immunol. 2026 Oct 5;17:1924000. doi: 10.3389/fimmu.2026.1924000 (PMC13677909; doi:10.3389/fimmu.2026.1924000)
Supplement: Supplementary file 1 [file DataSheet1.pdf]

Comparative profiling of MAIT cells across gastrointestinal cancers identifies conserved NK receptor dysregulation and HCC-specific defects in cytotoxicity and XBP1s signaling

Vanessa Proß[1], Annkathrin Helena Ruhm[1], Linda Marie Laura Thole[1], Laura Tóth[1], Gishnu Harikumar Parvathy[1], Pia Jäger[1], Christina Götz[2], Georg Damm[2], Daniel Seehofer[2], Christian Bayerl[3], Hermann Herbst[4], Alexander Macke[5], Verena Frese[5], Akio Sakaki[5], Marlene Hanisch-Hernandez[5], Friederike Rosseck[6], Sascha Treskatsch[7], Frank Friedersdorff[8], Christoph Treese[9], Severin Daum[9], Dominik Laue[10], Benjamin Ruf[11], Sören Lukassen[12], and Katja Kotsch, PhD[1*]

Supplementary Material

Supplementary Table 1. Antibodies for extracellular staining

Molecule	Clone	Fluorochrome	Manufacturer*	Cat. Number	RRID
CD3	SK7	PerCP/Cyanine5.5	BioLegend	344808	AB_10640736
CD4	SK3	BUV395	BD	563550	AB_2738273
CD8	SK1	APCeFluor780	Themo Fisher	47-0087-42	AB_2016684
CD14	M5E2	BV510	BioLegend	301842	AB_2561946
CD16	3G8	PE	BD	555407	AB_395807
CD16	NCAM16.2	BUV395	BD	563555	AB_2872414
CD19	H1B19	BV510	BioLegend	302242	AB_2561668
CD38	HIT2	BV605	BioLegend	303532	AB_2562915
CD56	5.1H11	BV785	BioLegend	362550	AB_2566059
CD56	REA	APC	Miltenyi	130-113-872	AB_2726365
CD69	FN50	AlexaFluor700	BioLegend	310922	AB_2726365
CD103	Ber-ACT8	PE	BioLegend	350206	AB_10641843
CD161	HP-3G10	PE/Dazzle 594	BioLegend	339940	AB_2565868
CD161	HP-3G10	BV421	BioLegend	339914	AB_2561421
L/D	-	BV510 (aqua)	BioLegend	423101	AB_3717566
NKG2D	1D11	BV421	BioLegend	320822	AB_2566511
NKp80	4A4.D10	PE/Cyanine7	Miltenyi	130-105-068	AB_2659831
NKp80	REA	PE	Miltenyi	130-112-779	AB_2653021

PD-1	EH12.1	BV711	BD	564017	AB_2738543
Vα7.2	3C10	APC	BioLegend	351708	AB_10933246
Vα7.2	3C10	PE/Cyanine7	BioLegend	351712	AB_2561994
2B4	C1.7	FITC	BioLegend	329506	AB_1279186

Supplementary Table 2. Antibodies for intracellular staining

Molecule	Clone	Fluorochrome	Manufacturer*	Cat. Number	RRID
Granzyme B	GB11	AlexaFluor700	BD	560213	AB_1645453
Granzyme B	GB11	FITC	BioLegend	515403	AB_2114575
IFNγ	4S.B3	eFluor450	Thermo Fisher	48-7319-42	AB_2043866
IFNγ	4S.B3	FITC	BioLegend	502507	AB_315232
IL-2	MQ1-17H12	BV605	BioLegend	500332	AB_2563877
IL-17	BL168	BV785	BioLegend	512338	AB_2566765
TNFα	MAb11	AlexaFluor700	BioLegend	502928	AB_2561315
TNFα	MAb11	BV711	BioLegend	502939	AB_2562740
XBP1s	Q3-695	PE	BD	562642	AB_2562740

* BioLegend, San Diego, USA
BD, Heidelberg, Germany
Thermo Fisher, Darmstadt, Germany
Miltenyi, Bergisch Gladbach, Germany
