# Supplementary material for: Comparative profiling of MAIT cells across gastrointestinal cancers identifies conserved NK receptor dysregulation and HCC-specific defects in cytotoxicity and XBP1s signaling
Source: Front Immunol. 2026 Oct 5;17:1924000. doi: 10.3389/fimmu.2026.1924000 (PMC13677909; doi:10.3389/fimmu.2026.1924000)
Supplement: Supplementary file 2 [file DataSheet2.pdf]

# Comparative profiling of MAIT cells across gastrointestinal cancers identifies conserved NK receptor dysregulation and HCC-specific defects in cytotoxicity and XBP1s signaling

Vanessa Proß[1], Annkathrin Helena Ruhm[1], Linda Marie Laura Thole[1], Laura Tóth[1], Gishnu Harikumar Parvathy[1], Pia Jäger[1], Christina Götz[2], Georg Damm[2], Daniel Seehofer[2], Christian Bayerl[3], Hermann Herbst[4], Alexander Macke[5], Verena Frese[5], Akio Sakaki[5], Marlene Hanisch-Hernandez[5], Friederike Rosseck[6], Sascha Treskatsch[7], Frank Friedersdorff[8], Christoph Treese[9], Severin Daum[9], Dominik Laue[10], Benjamin Ruf[11], Sören Lukassen[12], and Katja Kotsch, PhD[1*]

**Proß et al., Supplementary Figure 1**

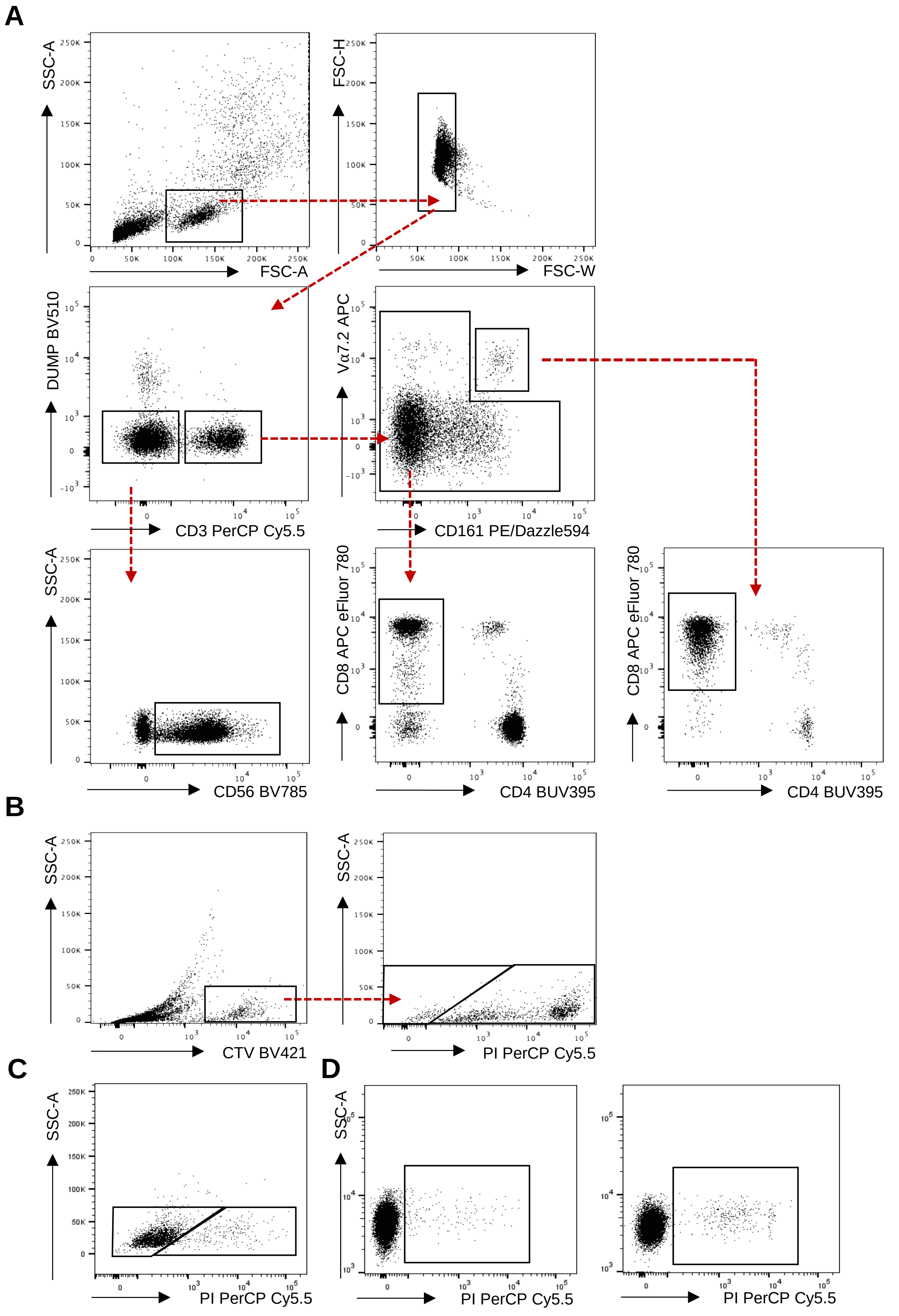


**Supplementary Figure 1. Gating strategy.** (A) Representative FACS plots for identification of MAIT cells, CD8⁺ T cells, and NK cells. (B) Definition of dead target cells (K562) in the cytotoxicity assay determinded by CTV staining and PI uptake. Cell death of K562 target (C) and effector (D) cells alone (left plot: MAIT cells, right plot: NK cells) after 12h of culture. CTV = CellTrace™ Violet; PI = Propidium iodide.

# Proß et al., Supplementary Figure 2

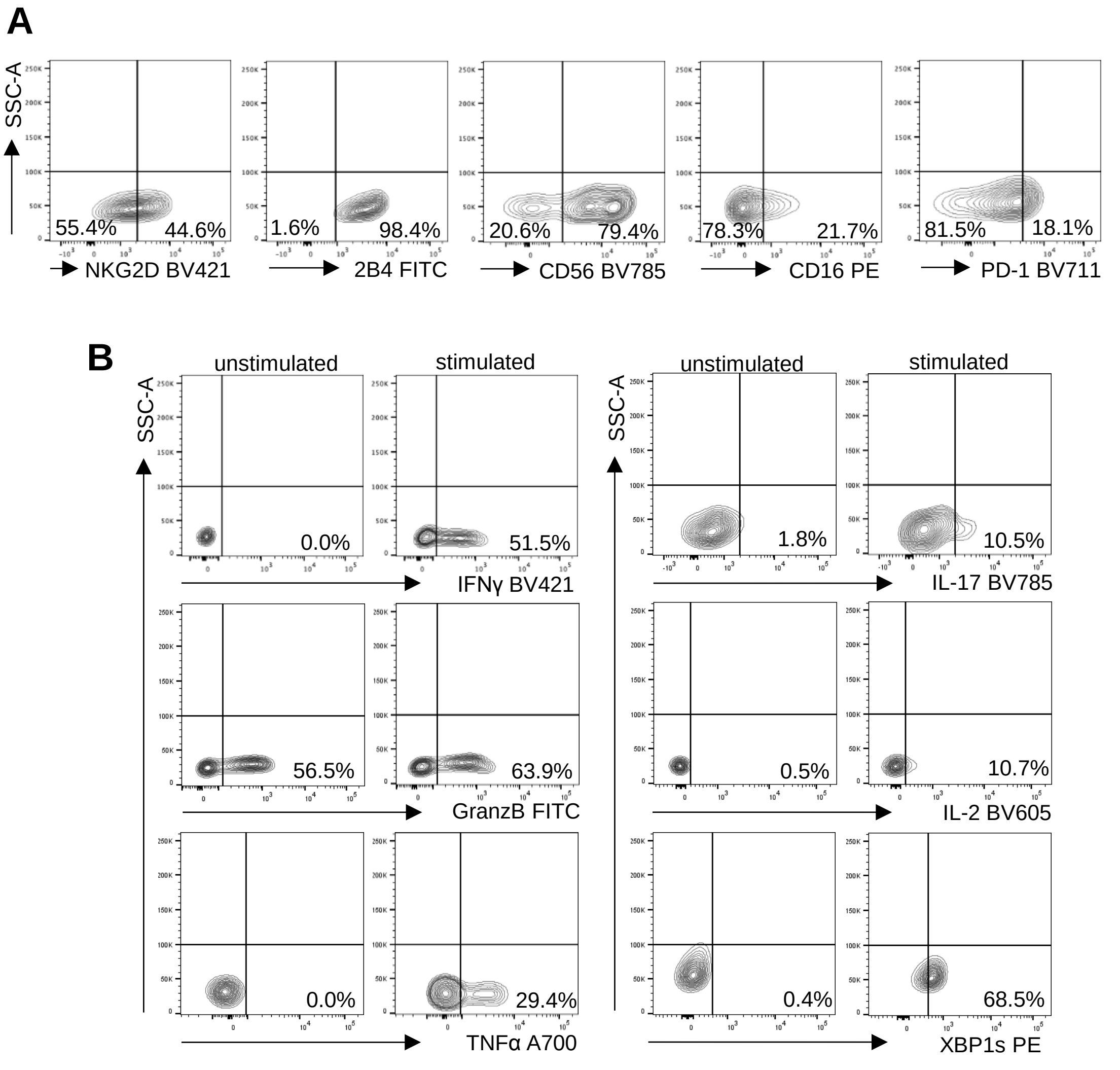


**Supplementary Figure 2. Representative flow cytometry dot plots.** (A) Gating for receptor expression. (B) Cytokine and XBP1s expression pre-gated on MAIT cells.

# Proß et al., Supplementary Figure 3

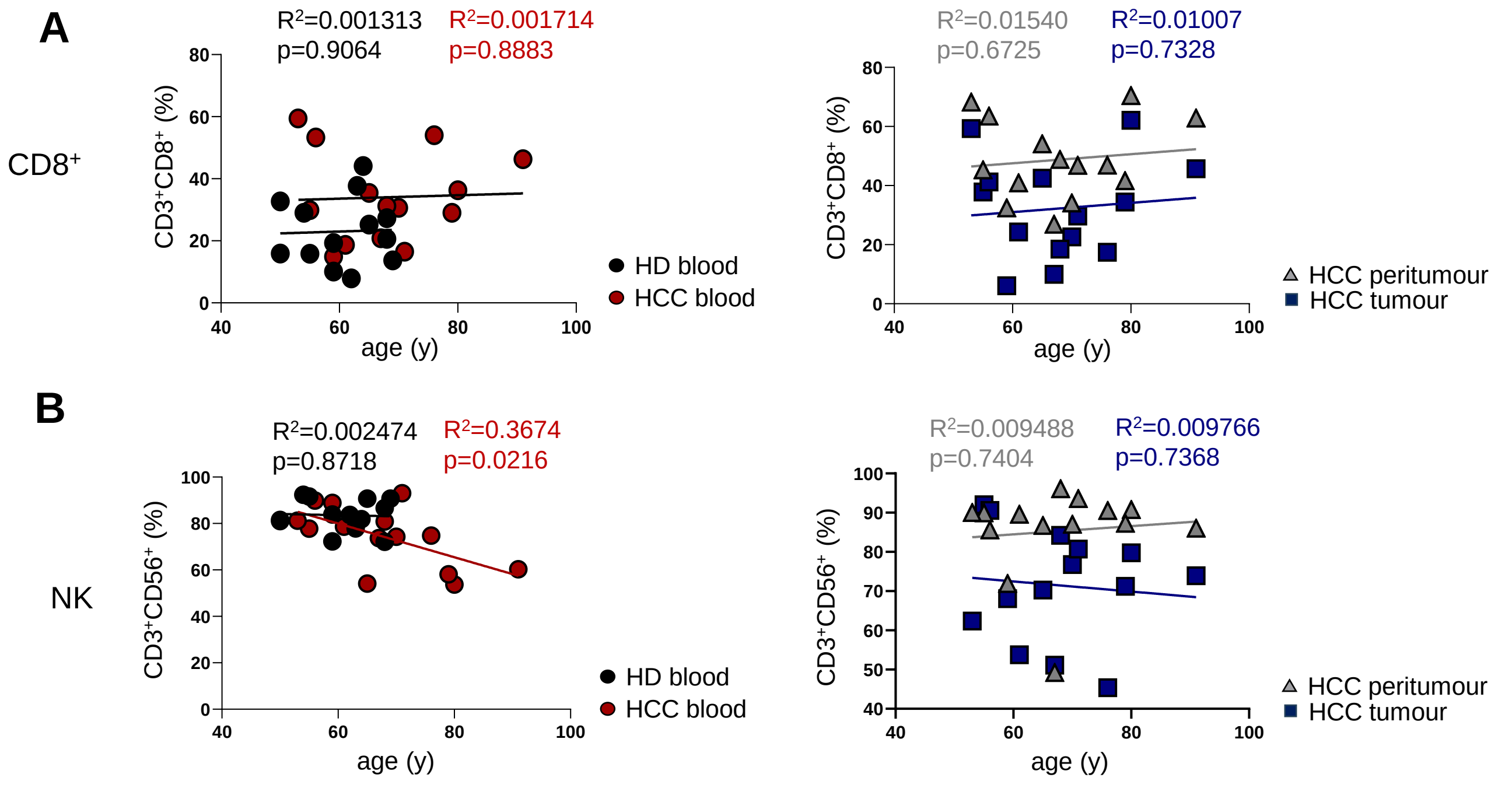


**Supplementary Figure 3. Correlation analyses with age.** Correlation of blood (derived from age-matched healthy donors and HCC tumour patients) and tissue (derived from HCC peritumour and tumour) to determine the frequencies of conventional $CD8^+$ T cells ($CD8^+$) (A) and NK cells (B) in relation to age. HD blood: n = 13; HCC blood: n = 14; HCC peritumour: n = 14; HCC tumour: n = 14; simple linear regression.

# Proß et al., Supplementary Figure 4

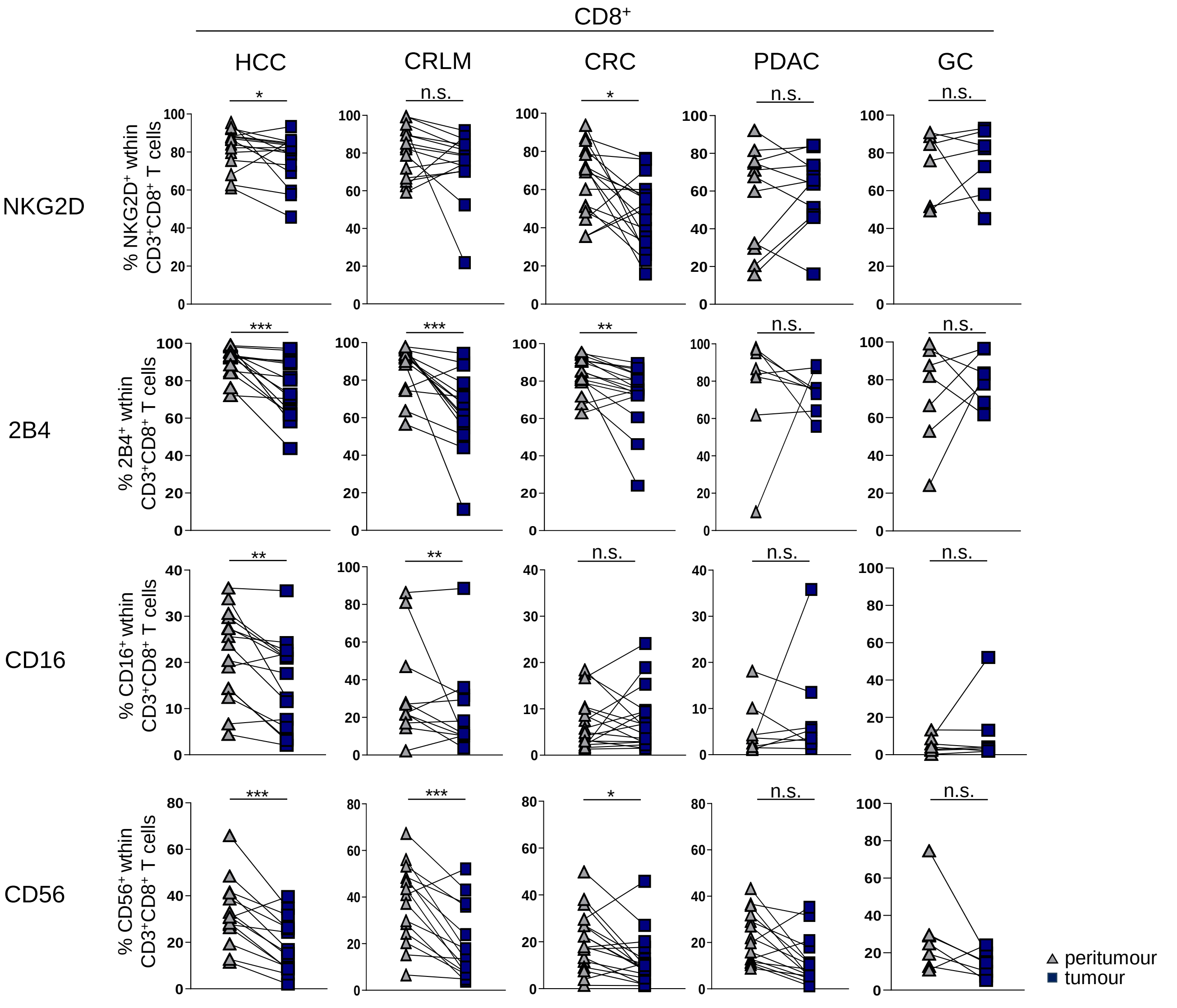


**Supplementary Figure 4. Phenotype of tumour-derived CD8⁺ T cells.** Frequencies of NKG2D⁺, 2B4⁺, CD56⁺, and CD16⁺ of CD8⁺ T cells derived from paired peritumour and tumour tissues. HCC: n = 14; CRLM: n = 15; CRC: n = 17; PDAC: n = 11; GC: n = 7. Non-significant (n.s.): $p>0.05$; *$p<0.05$; **$p<0.01$; ***$p<0.001$; calculated by two-tailed paired t-test or two-tailed Wilcoxon matched-pairs signed rank test.

**Proß et al., Supplementary Figure 5**

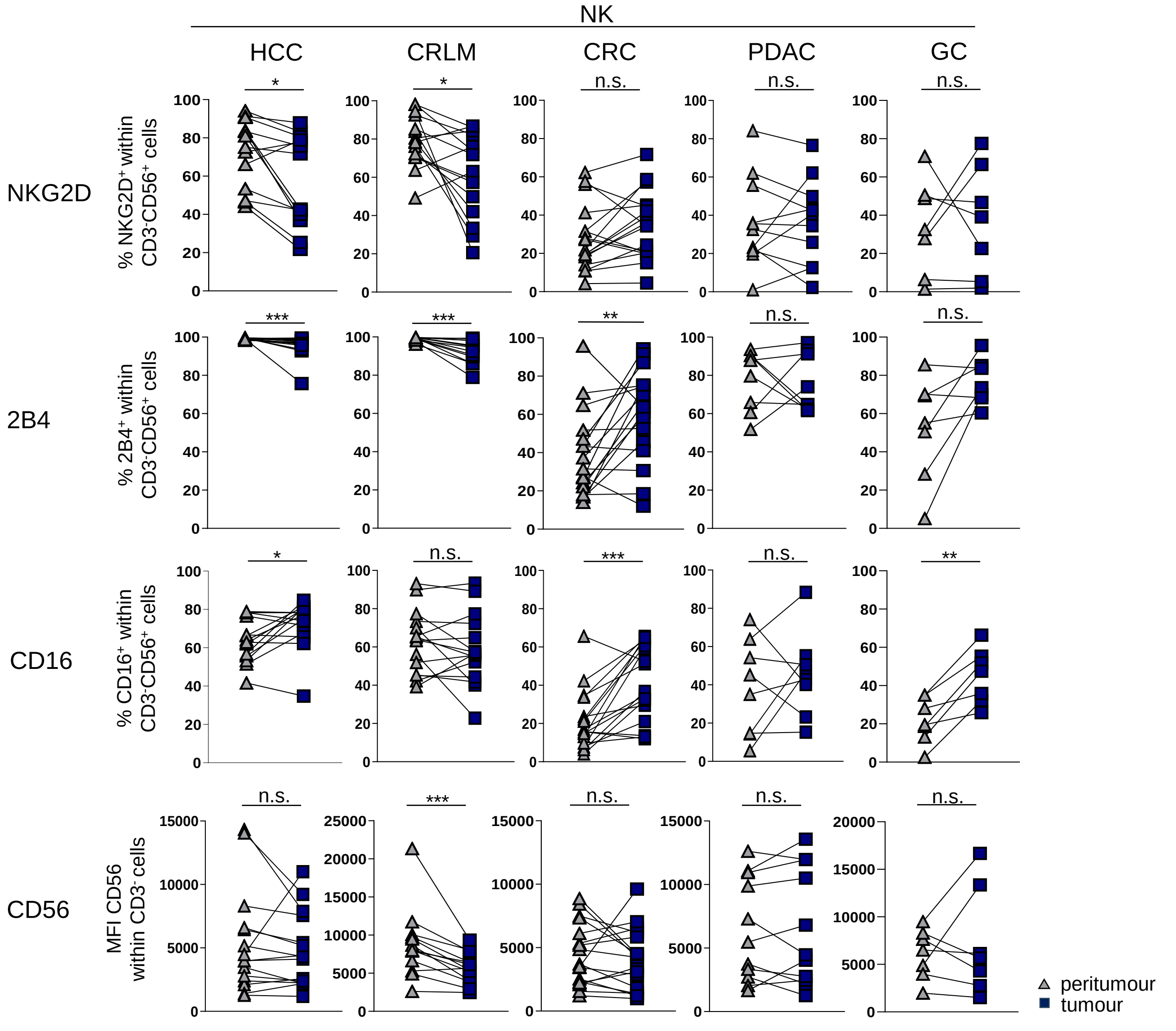


**Supplementary Figure 5. Phenotype of tumour-derived NK cells.** Frequencies of NKG2D+, 2B4+, CD16+ and MFI CD56 of NK cells derived from paired peritumour and tumour tissues. HCC: n = 14; CRLM: n = 15; CRC: n = 17; PDAC: n = 11; GC: n = 7. Non-significant (n.s.): p>0.05; *p<0.05; **p<0.01; ***p<0.001; calculated by two-tailed paired t-test or two-tailed Wilcoxon matched-pairs signed rank test.

# Proß et al., Supplementary Figure 6

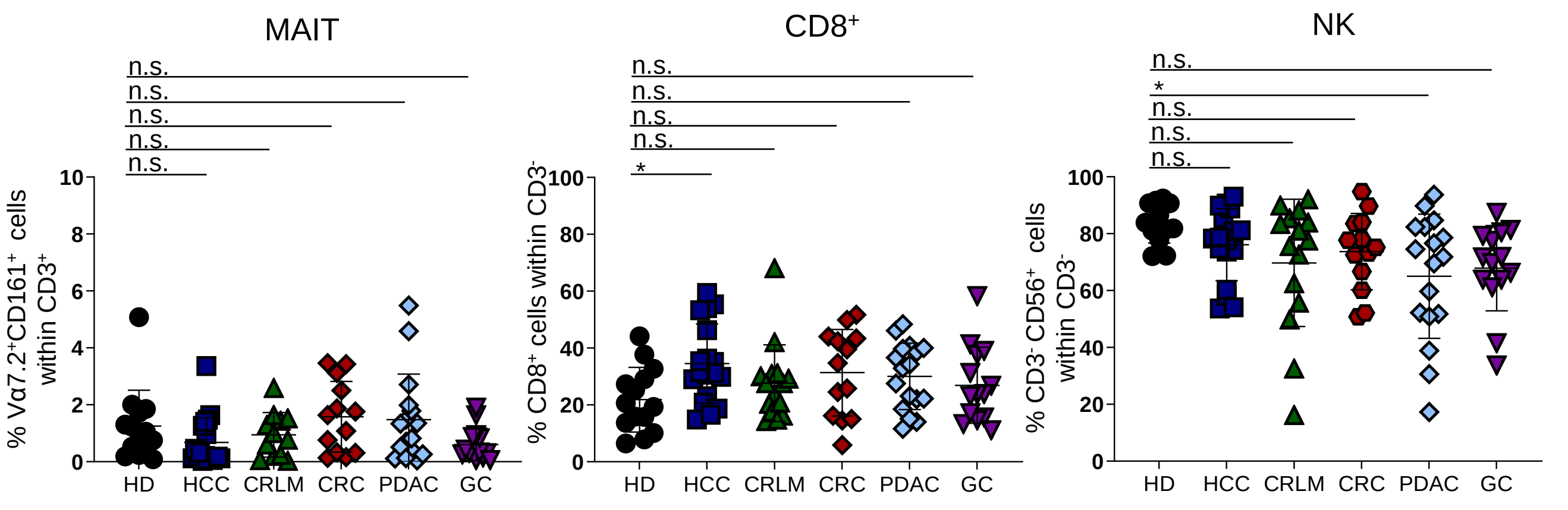


**Supplementary Figure 6. Blood-derived cell frequencies.** Frequencies of blood-derived MAIT cells, $CD8^+$ T cells, and NK cells from various tumour entities. HD: n = 14; HCC: n = 18; CRLM: n = 16; CRC: n=14; PDAC: n = 17; GC: n = 14. Non-significant (n.s.): p>0.05; *p<0.05; **p<0.01; ***p<0.001; calculated by using a Kruskal–Wallis test or a one-way ordinary ANOVA.

# Proß et al., Supplementary Figure 7

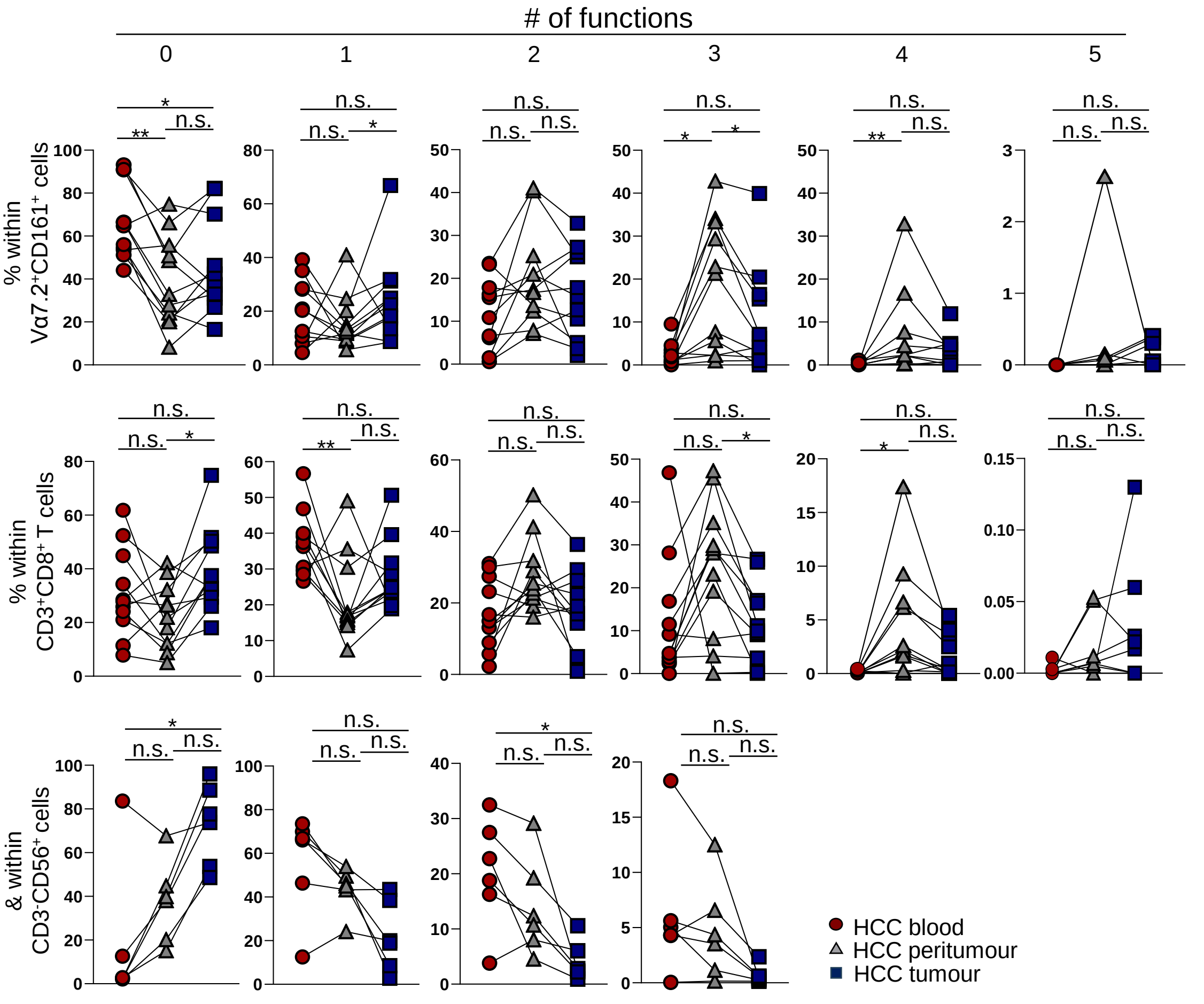


**Supplementary Figure 7. Polyfunctional effector responses.** (A) Frequencies of polyfunctional MAIT cells, $CD8^+$ T and NK cells derived from blood and HCC peritumoural and tumour tissues. HCC, n = 11–12. # of functions indicates the number of cytokines simultaneously expressed. Non-significant (n.s.): $p > 0.05$; * $p < 0.05$; ** $p < 0.01$; *** $p < 0.001$; calculated by using a Kruskal–Wallis test or a one-way ordinary ANOVA.

**Proß et al., Supplementary Figure 8**

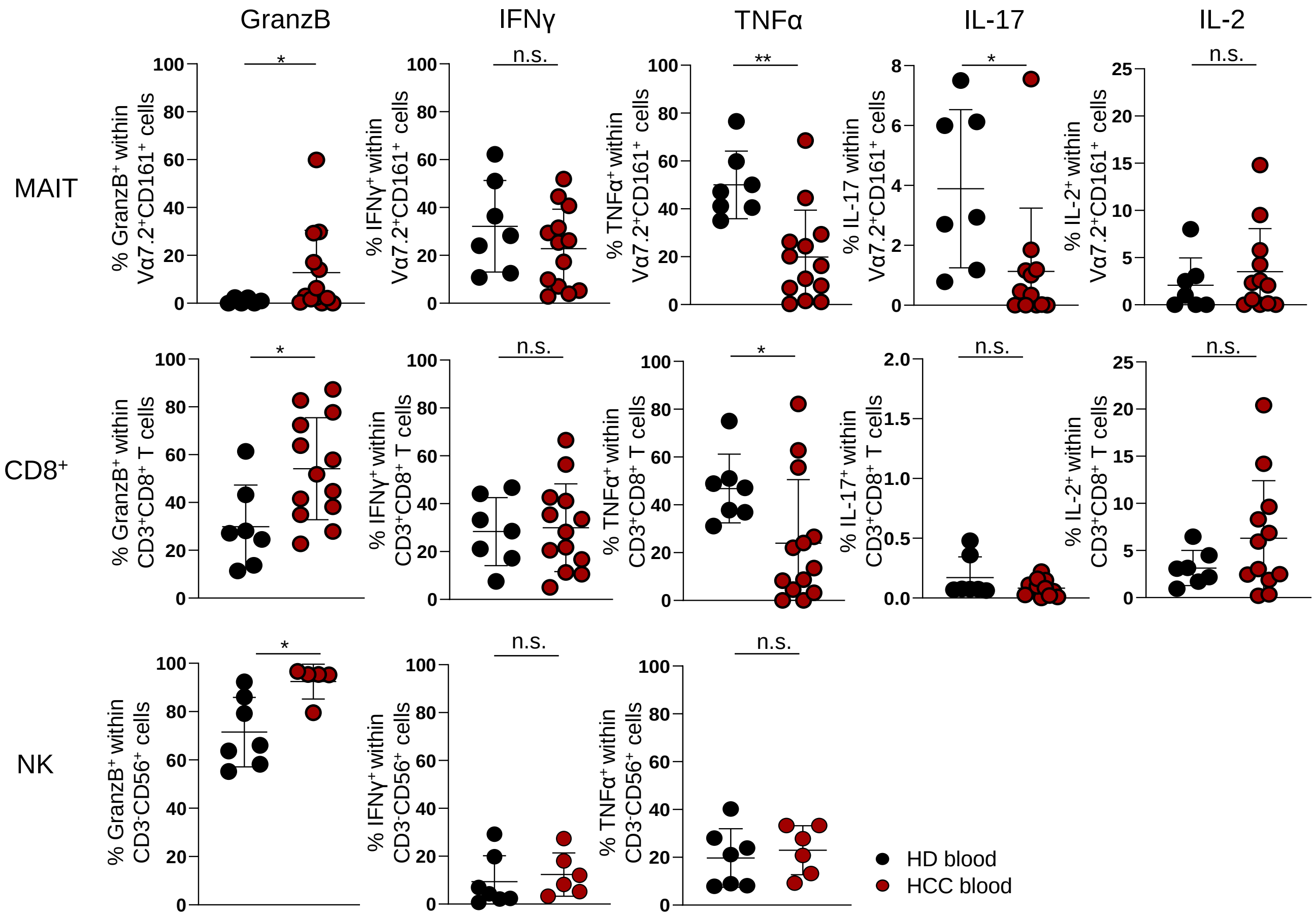


**Supplementary Figure 8. Peripheral MAIT cell effector profile in HCC patients and HD**. Frequencies of Granzyme B (GranzB), IFNγ, TNFα, IL-17 and IL-2 in blood-derived MAIT cells and for comparison reasons in blood-derived CD8$^+$ T and NK cells (HCC: n = 13) were compared to those in blood from age-matched healthy donors (n = 7). Non-significant (n.s.): $p > 0.05$; * $p < 0.05$; ** $p < 0.01$; *** $p < 0.001$; calculated by using a two-tailed paired t-test or a two-tailed Wilcoxon matched-pairs signed-rank test.

**Proß et al., Supplementary Figure 9**

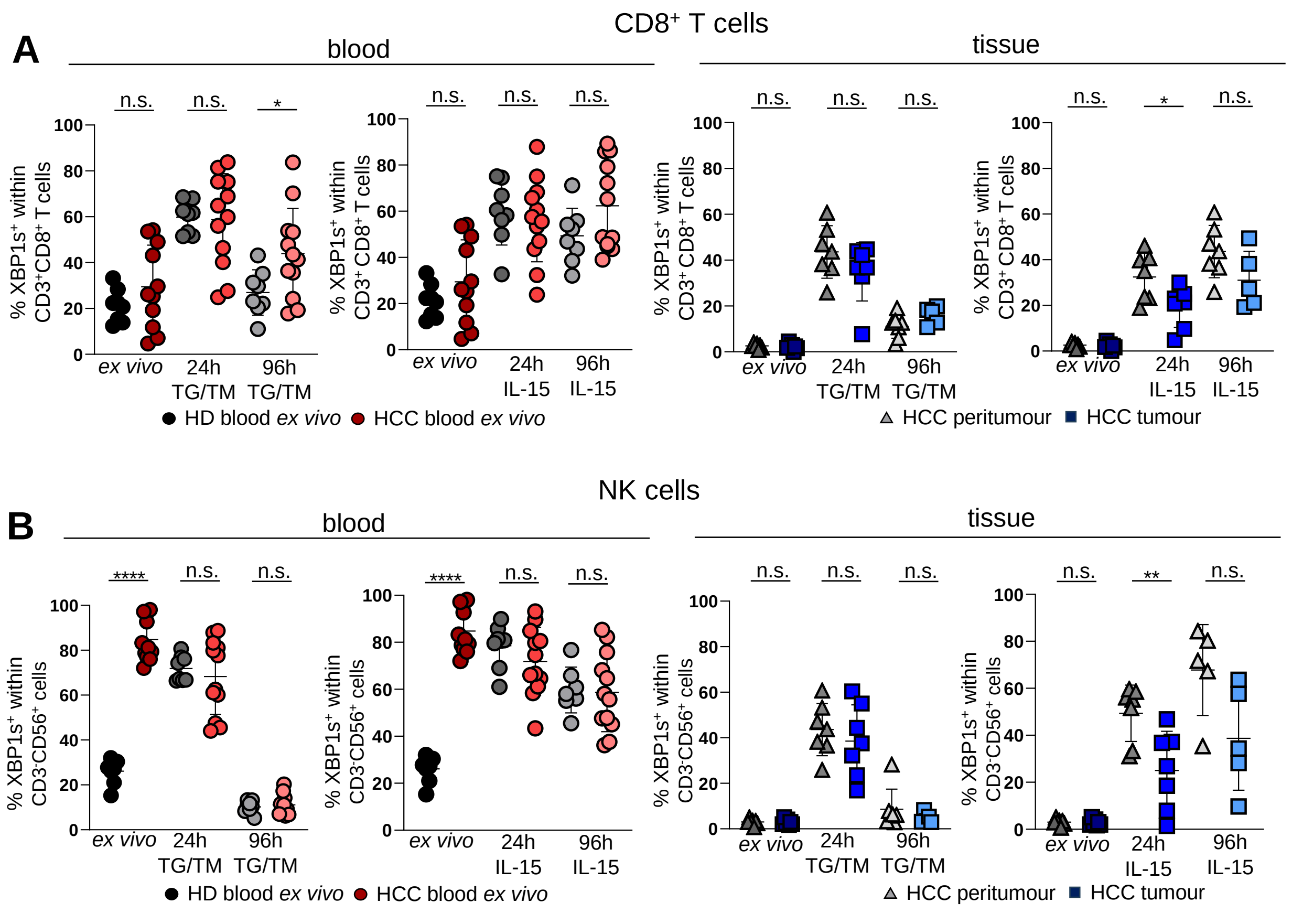


**Supplementary Figure 9. XBP1s activation in peripheral and tissue-derived CD8⁺ T cells and NK cells.** (A) Frequencies of XBP1s⁺ CD8⁺ T cells and (B) NK cells in peripheral blood, age-matched healthy donors (HD: n=8) and in tissue-derived (peritumoral and tumour) cells from patients with HCC (n = 6-10/tissue) analysed *ex vivo* or following stimulation with IL-15 or the ER stress inducers TG and TM for 24 or 96 hours. Non-significant (n.s.): $p > 0.05$; * $p < 0.05$; ** $p < 0.01$; *** $p < 0.001$, calculated by using a two-tailed paired t-test or a two-tailed Wilcoxon matched-pairs signed-rank test.
